# Selective inhibition of mTORC1 in tumor vessels increases antitumor immunity

**DOI:** 10.1172/jci.insight.139237

**Published:** 2020-08-06

**Authors:** Shan Wang, Ariel Raybuck, Eileen Shiuan, Sung Hoon Cho, Qingfei Wang, Dana M. Brantley-Sieders, Deanna Edwards, Margaret M. Allaman, James Nathan, Keith T. Wilson, David DeNardo, Siyuan Zhang, Rebecca Cook, Mark Boothby, Jin Chen

**Affiliations:** 1 Veterans Affairs Medical Center, Tennessee Valley Healthcare System, Nashville, Tennessee, USA.; 2 Division of Rheumatology and Immunology and; 3 Department of Pathology, Microbiology and Immunology, Vanderbilt University Medical Center, Nashville, Tennessee, USA.; 4 Program in Cancer Biology, School of Medicine, Vanderbilt University, Nashville, Tennessee, USA.; 5 Department of Biological Sciences, Harper Cancer Research Institute, University of Notre Dame, South Bend, Indiana, USA.; 6 Vanderbilt-Ingram Cancer Center and; 7 Division of Gastroenterology, Hepatology, and Nutrition, Vanderbilt University Medical Center, Nashville, Tennessee, USA.; 8 Department of Medicine, University of Cambridge, Cambridge, United Kingdom.; 9 Department of Medicine, Washington University School of Medicine in St. Louis, St. Louis, Missouri, USA.; 10 Department of Cell and Developmental Biology, School of Medicine, Vanderbilt University, Nashville, Tennessee, USA.

**Keywords:** Immunology, Oncology, Cancer immunotherapy, endothelial cells

## Abstract

A tumor blood vessel is a key regulator of tissue perfusion, immune cell trafficking, cancer metastasis, and therapeutic responsiveness. mTORC1 is a signaling node downstream of multiple angiogenic factors in the endothelium. However, mTORC1 inhibitors have limited efficacy in most solid tumors, in part due to inhibition of immune function at high doses used in oncology patients and compensatory PI3K signaling triggered by mTORC1 inhibition in tumor cells. Here we show that low-dose RAD001/everolimus, an mTORC1 inhibitor, selectively targets mTORC1 signaling in endothelial cells (ECs) without affecting tumor cells or immune cells, resulting in tumor vessel normalization and increased antitumor immunity. Notably, this phenotype was recapitulated upon targeted inducible gene ablation of the mTORC1 component Raptor in tumor ECs (Raptor^ECKO^). Tumors grown in Raptor^ECKO^ mice displayed a robust increase in tumor-infiltrating lymphocytes due to GM-CSF–mediated activation of CD103^+^ dendritic cells and displayed decreased tumor growth and metastasis. GM-CSF neutralization restored tumor growth and metastasis, as did T cell depletion. Importantly, analyses of human tumor data sets support our animal studies. Collectively, these findings demonstrate that endothelial mTORC1 is an actionable target for tumor vessel normalization, which could be leveraged to enhance antitumor immune therapies.

## Abstract 2

Inhibition of mTORC1 or endothelial-specific deletion of Raptor improves anti-tumor immunity and enhances adoptive T cell therapy.

## Introduction

A rapidly growing tumor requires blood vessels to support the increased demands for nutrients and oxygen. Cancer cells produce angiogenic factors that direct rapid blood vessel growth. However, unlike vessels in healthy tissues, tumor blood vessels are highly dysfunctional, characterized by chaotic networks of leaky, tortuous, and uneven endothelial tubules without pericyte support (reviewed in refs. 1, 2). These vascular abnormalities cause sluggish blood flow, vascular leakiness, and poor perfusion in tumors. Given that many anticancer treatments are delivered to the tumor through the circulation, the inherently diminished function of the tumor vasculature may limit drug delivery to tumors, thus diminishing the efficacy of treatments (3). Further, immune responses and leukocyte trafficking may be affected by poor tumor perfusion, as well as by the hypoxic microenvironment created by the faulty vessels (4, 5). Constant vessel remodeling in tumors also contributes to spontaneous hemorrhage and increased interstitial fluid pressure, which blocks drug delivery and enhances a malignant tumor phenotype, including metastasis (6). As such, the abnormal nature of the tumor vasculature enhances tumor metastasis, immune escape, and poor therapeutic response. Traditional antiangiogenic therapy blocks tumor vessel growth, which can limit the growth of many tumors. However, the abolition of angiogenesis further heightens hypoxia and interstitial pressure within the tumor, thus enhancing the escape of cancer cells via invasion and metastasis in animal models (7, 8). An emerging concept for improved therapy is to find ways to improve the functionality of, or to “normalize,” tumor blood vessels (9–11). Vessel normalization increases vessel pericyte coverage, reduces vessel leakiness, improves tumor perfusion, and decreases tumor hypoxia.

Notably, recent evidence suggests that tumor vessel normalization may enhance infiltration of the tumor parenchyma by leukocytes (1, 12–15), a discovery consistent with observations that blood vessels regulate leukocyte trafficking in different tissues (16, 17). Importantly, T cell exclusion from the tumor parenchyma limits tumor response to immunotherapy. Conversely, leukocytes within tumors may influence the integrity of the tumor vasculature. For example, T helper type 1 (Th1) CD4^+^ T cells, M1-like tumor-associated macrophages (TAMs), and eosinophils each enhance tumor vessel normalization (13, 18, 19). This immune-vascular crosstalk suggests that tumor vessel normalization may contribute substantially to tumor immunology, particularly in the context of immunotherapy.

The serine/threonine kinase mTOR is a central member of a multi-subunit signaling complex that integrates environmental cues to regulate proliferation, survival, and metabolic homeostasis in many cell types (20, 21). The importance of mTOR signaling in tumor cells has been extensively studied (20, 21), but relatively little is known about mTOR signaling in tumor blood vessels. The mTOR kinase acts in 2 functionally distinct complexes with shared (e.g., mTOR and mTOR associated protein, LST8 homolog) and unique (e.g., Raptor in mTORC1, Rictor in mTORC2) components. mTORC1 phosphorylates several substrates involved in protein translation and cellular proliferation, including ribosomal protein S6 kinase-1 (S6K1). Interestingly, S6K1 knockdown in vascular endothelial cells (ECs) inhibits angiogenesis in vivo (22), suggesting that signaling through the mTORC1 pathway might affect vascular ECs. Conversely, constitutively active S6K1 expression increases reactive oxygen species (ROS) generation and NADPH oxidase (NOX1 and NOX2) mRNA levels in EC cultures (23). Further, aberrant mTORC1 activation in ECs promotes angiogenesis (24), driving formation and progression of lymphangiosarcoma (25). Together, these studies implicate mTORC1 signaling in growth and/or homeostasis of the tumor vasculature, but this hypothesis has not yet been tested in the context of tumor vasculature.

In this report, we demonstrate that EC-specific deletion of Raptor (Raptor^ECKO^), a unique component of mTORC1, caused tumor blood vessel normalization and decreased tumor growth and lung metastasis. Tumors with Raptor/mTORC1-deficient ECs harbored increased tumor-infiltrating lymphocytes (TILs) with enhanced cytotoxic T cell activation markers, increased CD103^+^ DCs, and elevated GM-CSF. In vivo neutralization of either GM-CSF or T cells partially rescued phenotypes in Raptor^ECKO^ tumors, suggesting that tumor growth inhibition was due, at least in part, to an enhanced GM-CSF–directed immune response. In human tumors, expression of vessel normalization markers negatively correlated with endothelial expression of mTORC1/RAD001-sensitive genes, while GM-CSF levels positively correlated with markers of immune cells, CD103^+^ DCs, and cytotoxic gene signature. Importantly, low-dose RAD001, an mTORC1 inhibitor, induced selective inhibition of mTORC1 signaling in tumor ECs, promoted vessel normalization, increased TILs, and improved adoptive T cell therapy. Taken together, our findings suggest endothelial mTORC1 inhibition is a novel approach to normalize tumor blood vessels and enhance antitumor immunity.

## Results

### Low-dose RAD001 selectively inhibits mTORC1 signaling in tumor endothelium and suppresses tumor growth.

**Figure 1:**
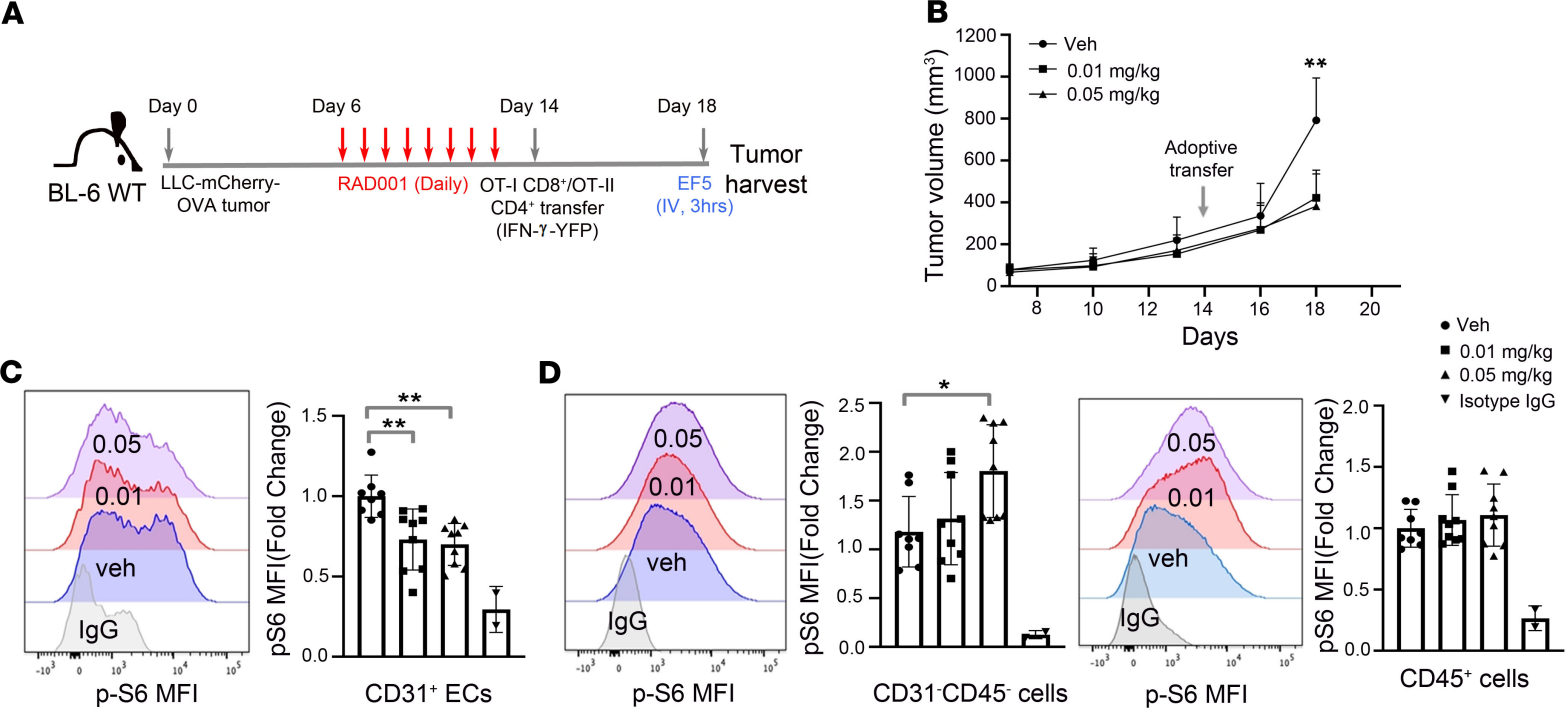
Low-dose RAD001 selectively inhibits mTORC1 signaling in tumor endothelium and suppresses tumor growth. (**A**) Schematic diagram showing the experimental design with LLC-HRE-mCherry-OVA tumor cell implantation, RAD001 treatment, adoptive T cell transfer, and EF5 intravenous injection. (**B**) Growth curves of LLC-HRE-mCherry-OVA tumors treated with a low dose of RAD001. *n* = 14–16 mice per group. *P* values were determined by Student’s *t* tests comparing vehicle- and RAD001-treated groups at day 18. (**C** and **D**) Flow cytometric analysis showing low-dose RAD001 treatment decreased p-S6 level in CD45^–^CD31^+^ tumor-associated ECs (**C**) but not in LLC tumor cells (CD45^–^CD31^–^) and immune cells (CD45^+^) (**D**). MFI, mean fluorescence intensity. All data are presented as mean ± SD, and *P* values were determined by 1-way ANOVA with post hoc Tukey’s correction for multiple comparisons. **P ≤ 0.01, *P ≤ 0.05.

mTORC1 is a signaling node downstream of multiple angiogenic factors. mTORC1 inhibitors are FDA approved but conventionally used at doses that are immunosuppressive. However, recent studies and clinical trials discovered that low-dose mTORC1 inhibitors, such as RAD001/everolimus, inhibit mTORC1 signaling but are not immunosuppressive (26, 27). To determine the effect of low-dose RAD001 on tumor blood vessels and tumor growth, we implanted Lewis lung carcinoma (LLC) tumor cells containing expression of hypoxia responsive element–driven (HRE-driven) mCherry and ovalbumin (OVA), referred to as LLC-HRE-mCherry-OVA, into C57BL/6 recipient mice (Figure 1A). Six days after LLC tumor implantation, mice were given RAD001 daily at 0.01 mg/kg or 0.05 mg/kg or vehicle control for 8 days, followed by adoptive transfer of preactivated Thy1.1^+^ (OT-I) CD8^+^ and OT-II CD4^+^ T cells expressing the IFN-γ–yellow fluorescent protein (YFP) reporter (28–30). Because T cells harvested from OT-I or OT-II transgenic mice express the T cell receptor (TCR) that specifically recognizes the OVA-derived peptide antigen bound to MHC-I or MHC-II, this approach allows for a detailed analysis of tumor antigen–directed T cell responses. As shown in Figure 1B, treatment with both low doses of RAD001 significantly reduced tumor volume at day 18, 4 days after T cell adoptive transfer, indicating that low-dose RAD001 improved adoptive T cell therapy. Phospho–flow cytometry analysis revealed that low-dose RAD001 treatment inhibited p-S6, a downstream target of mTORC1 signaling, in CD31^+^ ECs (Figure 1C), but not in CD45^–^CD31^–^ populations (including tumor cells) and CD45^+^ immune cells (Figure 1D), suggesting that low doses of RAD001 primarily target ECs within the tumor microenvironment.

### Loss of Raptor/mTORC1 in ECs reduces tumor growth and metastasis.

To investigate the role of mTORC1 in vascular ECs genetically, we crossed mice harboring floxed *Raptor* alleles (Raptor^fl/fl^, referred to as Raptor^WT^) with mice expressing tamoxifen-inducible Cre recombinase (Cre^ER^) under the control of the *Cdh5*/vascular endothelial-cadherin (VE-Cad) gene promoter, thus generating mice with inducible EC-specific loss of Raptor (referred to as Raptor^ECKO^) following tamoxifen treatment (Supplemental Figure 1A; supplemental material available online with this article; https://doi.org/10.1172/jci.insight.139237DS1). Raptor^ECKO^ mice were viable and healthy before and following tamoxifen treatment. Previous studies suggest that lung vascular ECs with Raptor knockout are viable and competently form tubules in culture (31), suggesting that Raptor/mTORC1 is not required for the survival of vascular ECs in adult animals.

**Figure 2:**
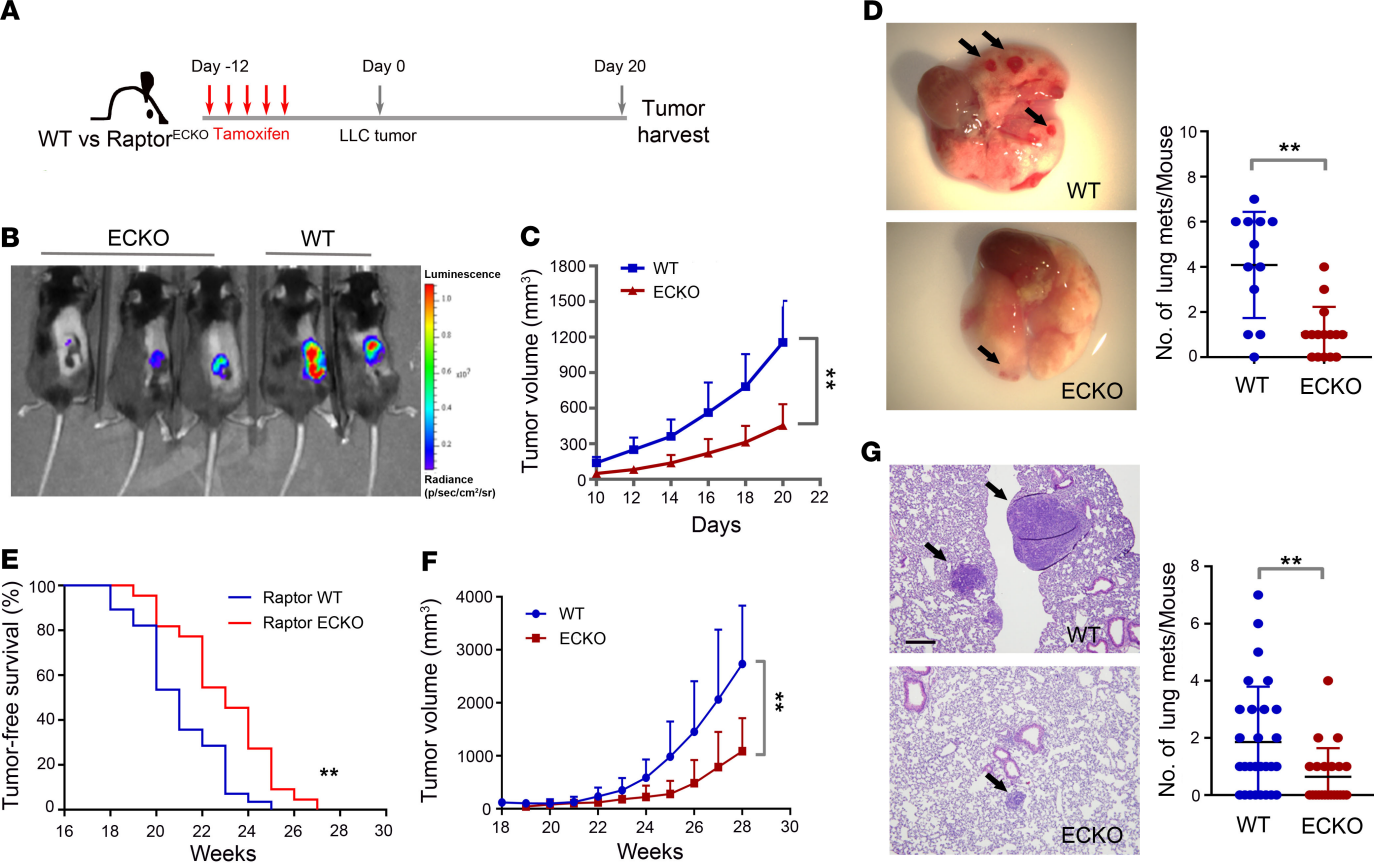
Raptor/mTORC1 loss in tumor endothelium decreases tumor growth and metastasis. (**A**) Schematic diagram showing the experimental procedure of tamoxifen treatment and subcutaneous implantation of LLC tumor nodules. (**B**) Representative image of bioluminescence signal from LLC tumors on WT control and Raptor^ECKO^ mice. (**C**) Growth curves of LLC tumors on WT control and Raptor^ECKO^ mice. Tumors were measured by a caliper every other day from day 10 through 20 after tumor implantation. *n* = 12 to 15 mice per group. **P ≤ 0.01, 2-way ANOVA. (**D**) Representative images of the lungs harvested from WT and Raptor^ECKO^ mice after 20 days of LLC tumor implantation. Arrows indicate metastatic foci on the surface of lungs, which were quantified. (**E**) Disease-free survival of spontaneous MMTV-PyMT tumors against age (weeks). *n* = 22 to 28 mice per group. **P ≤ 0.01. Statistical analysis was performed using log-rank test. (**F**) Growth curves of spontaneous MMTV-PyMT tumors on WT control and Raptor^ECKO^ mice. **P ≤ 0.01, 2-way ANOVA. (**G**) Representative H&E staining of lungs harvested from WT and Raptor^ECKO^/*MMTV-PyMT* mice. Arrows indicate metastatic foci within the lungs, which were quantified. Scale bar: 200 μm. Unless indicated, all data are presented as mean ± SD, and *P* values were determined by 2-tailed unpaired Student’s 2-tailed *t* test. **P ≤ 0.01.

To investigate the contribution of mTORC1 specifically within tumor vasculature, we implanted LLC tumor nodules into tamoxifen-treated WT or Raptor^ECKO^ recipient mice and measured tumor size over a time course (Figure 2A). These LLC tumor nodules were propagated through serial in vivo transplantation and retained their ability to metastasize in 100% of animals (32). Measurements of tumor burden using bioluminescence (Figure 2B) or a caliper-based method (Figure 2C) demonstrated significantly decreased tumor growth in Raptor^ECKO^ mice as compared with controls. IHC measurements of proliferating cell nuclear antigen revealed decreased tumor cell proliferation in Raptor^ECKO^ tumors (Supplemental Figure 1B), while TUNEL analysis revealed increased tumor cell death (Supplemental Figure 1C). Lungs harvested at the study endpoint (~3 weeks after tumor implantation) were assessed for surface metastases, revealing substantially decreased metastases in Raptor^ECKO^ mice as compared with WT mice (Figure 2D). To rule out the influence of tumor size on the metastatic potential of tumors grown in Raptor^ECKO^ mice, we repeated these studies with a modified approach, removing primary LLC tumors when tumor volume reached 500 mm^3^, and then assessed lung metastasis 12 days following tumor resection (Supplemental Figure 1D). Measurements of lung bioluminescence (Supplemental Figure 1, E and F), as well as counting of lung surface metastatic lesions (Supplemental Figure 1G), showed a diminished burden of lung metastases in Raptor^ECKO^ mice as compared with controls. These studies confirm that mTORC1 signaling within tumor vasculature supports tumor cell growth, survival, and metastasis.

To complement tumor allograft studies, we analyzed the EC-specific Raptor/mTORC1 loss in the transgenic *MMTV-PyMT* spontaneous mammary tumor model (33), using Raptor^ECKO^ mice crossed with *MMTV-PyMT* mice (Raptor^ECKO^ PyMT). At 8 weeks of age, female Raptor^WT^ PyMT and Raptor^ECKO^ PyMT mice were treated with tamoxifen to induce irreversible *Raptor* loss from vascular ECs. Tumor burden was monitored weekly beginning at 18 weeks of age. Notably, mammary tumor latency was delayed (Figure 2E), while tumor growth was markedly reduced (Figure 2F) in tamoxifen-treated Raptor^ECKO^ PyMT mice as compared with tamoxifen-treated controls. Further, lung metastasis was significantly inhibited in 28-week-old tamoxifen-treated Raptor^ECKO^ PyMT mice as compared with age-matched controls (Figure 2G). These data confirm findings using the LLC allografted tumor model and suggest that Raptor/mTORC1 loss from tumor blood vessels inhibits tumor growth and lung metastasis.

### Selective inhibition of mTORC1 in ECs decreases angiogenic sprouts and normalizes tumor blood vessels.

**Figure 3:**
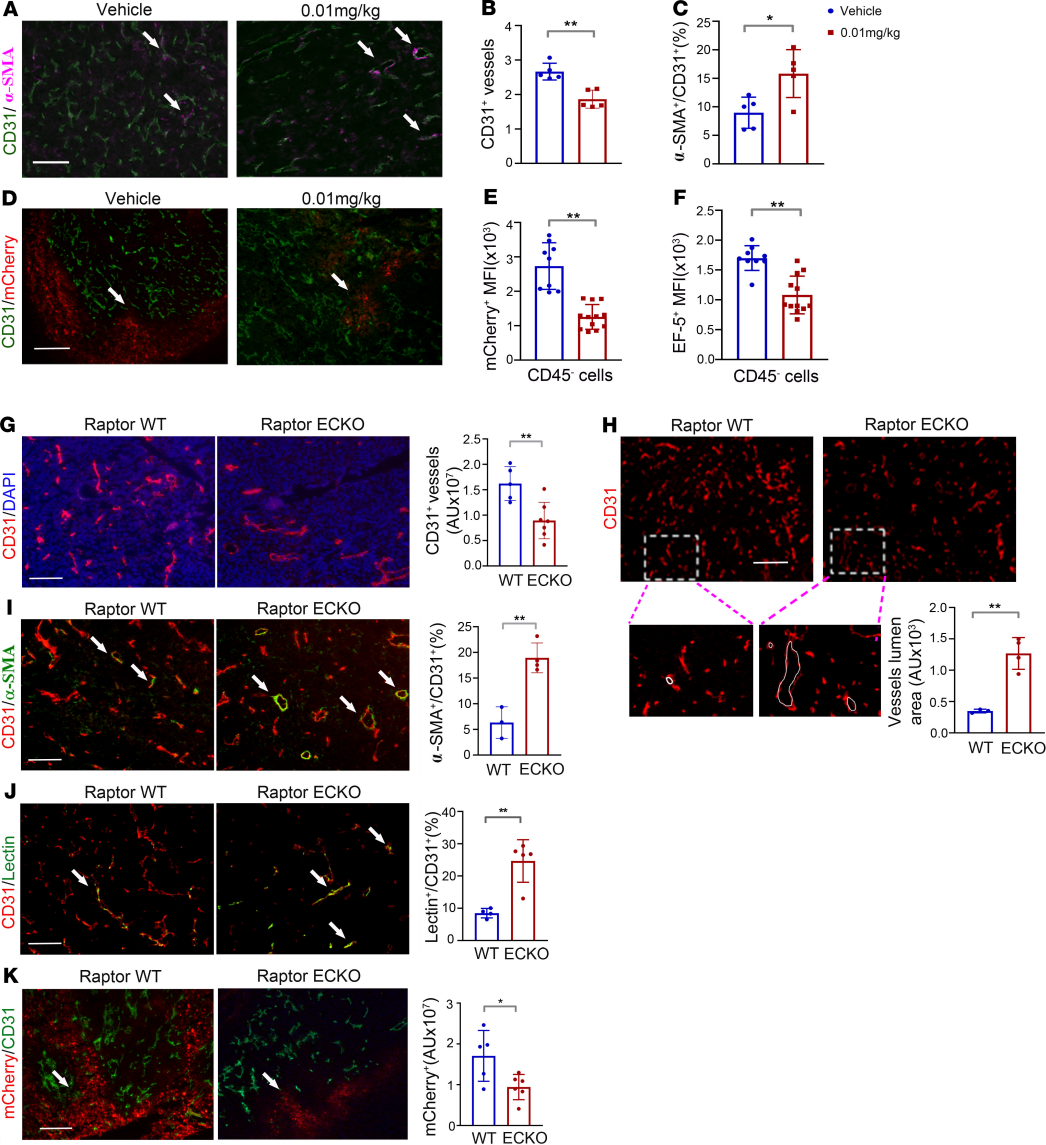
Selective inhibition of mTORC1 in endothelium normalizes tumor blood vessels. (**A**) Representative images of CD31^+^ (shown in green, EC marker) and α-SMA (shown in magenta, pericyte marker) costaining in LLC-HRE-mCherry-OVA tumors treated with low-dose RAD001. Arrows indicate colocalization of CD31^+^ and α-SMA. Scale bar: 100 μm. (**B**) Tumor vessel density was quantified as CD31^+^ area/field in LLC-HRE-mCherry-OVA tumors. (**C**) Pericyte coverage on tumor blood vessels was quantified and presented as percentage of α-SMA^+^CD31^+^ vessels. (**D**) Representative images of mCherry expression (red) in LLC-HRE-mCherry-OVA tumors treated with low-dose RAD001. Tumor vessels were assessed by CD31 staining (green). Arrows indicate mCherry^+^ hypoxic area. Scale bar: 50 μm. (**E** and **F**) Hypoxic regions in LLC-HRE-mCherry-OVA tumors were quantified by flow cytometry to assess the fluorescence intensity of mCherry^+^ (**E**) and EF5^+^ (**F**) in CD45^–^ tumor cells after RAD001 treatment. (**G**) Representative images and quantification of CD31^+^ blood vessels (red) in LLC tumors harvested from WT control and Raptor^ECKO^ mice. *n* = 5–7 mice per group. Scale bar: 100 μm. (**H**) Representative images and quantification of lumen size of CD31^+^ vessels from WT and Raptor^ECKO^ tumors. Zoomed-in images (original magnification, ×20) of dotted-line area are shown at the bottom. White solid lines mark lumen area in CD31^+^ vessels. *n* = 3 mice per group. Scale bar: 100 μm. (**I**) Costain of CD31^+^ (red) and α-SMA (green) in LLC tumors from WT control and Raptor^ECKO^ mice. Arrows indicate colocalization of CD31 and SMA. Pericyte coverage on tumor blood vessels was quantified and presented as percentage of α-SMA^+^CD31^+^ vessels. *n* = 3–4 mice per group. Scale bar: 100 μm. (**J**) Representative images showing lectin perfusion (green) in CD31^+^ tumor blood vessels (red). Arrows indicate lectin-perfused functional blood vessels. Vessel perfusion was quantified and presented as percentage of Lectin^+^CD31^+^/total CD31^+^ vessels. *n* = 5 mice per group. Scale bar: 100 μm. (**K**) Hypoxia was assessed by mCherry expression (red) in WT control and Raptor^ECKO^ tumors and quantified as mCherry^+^ intensity within tumors. *n* = 5–6 mice per group. Scale bar: 50 μm. AU, arbitrary units. All data are presented as mean ± SD. **P ≤ 0.01. *P ≤ 0.05, Student’s 2-tailed *t* test.

To determine the impact of Raptor/mTORC1 on tumor vasculature, we first assessed tumor microvessel density and morphology in situ using CD31 and smooth muscle actin (α-SMA), a pericyte marker, to visualize ECs in low-dose RAD001–treated LLC-HRE-mCherry-OVA tumors (Figure 3A). Treatment with low-dose RAD001 (0.01 mg/kg) reduced the density of CD31^+^ tumor vessels (Figure 3B) and induced an increase in pericyte coverage of tumor vessels, as measured by CD31/α-SMA costaining in tumors (Figure 3C), indicating an improvement in vessel maturation. Further, measurements of tumor hypoxia using the HRE-mCherry reporter (34) revealed that mCherry expression (Figure 3, D and E) was decreased in LLC-HRE-mCherry-OVA tumors after low-dose RAD001 treatment, and reduced hypoxia was confirmed by the staining of a hypoxic marker, EF5, on tumor cells (Figure 3F). Taken together, these data suggest that low-dose RAD001 preferentially inhibits mTORC1 signaling in ECs, leading to an increase in tumor vessel normalization.

To verify the role of endothelium-specific mTORC1 in vessel normalization, we next accessed tumor vasculature in LLC tumors grown on Raptor^WT^ and Raptor^ECKO^ mice. Consistent with our finding in low-dose RAD001 treatment, the density of CD31^+^ tumor vessels was decreased in tumors after Raptor/mTORC1 was deleted in vessels (Figure 3G). Morphometric analyses of vascular lumen area revealed increased lumen size of Raptor^ECKO^ tumor vessels compared with Raptor^WT^ tumor vessels (Figure 3H), consistent with the morphology of normalized blood vessels. In line with this observation, pericyte coverage of tumor vessels was increased in Raptor^ECKO^ tumors relative to WT tumors (Figure 3I). Further, perfusion of vasculature was increased in Raptor^ECKO^ tumors, as revealed by lectin perfusion assays (Figure 3J). Importantly, tumor cells expressing hypoxia-induced mCherry localized in areas distant from CD31^+^ vessels and mCherry^+^ intensity were significantly reduced in Raptor^ECKO^ samples as compared with Raptor^WT^ samples (Figure 3K), suggesting increased perfusion and oxygen delivery by vessels lacking mTORC1 signaling. However, loss of Raptor in tumor-free endothelium did not significantly change CD31^+^ vessel intensity or α-SMA^+^ pericyte coverage in normal tissues (Supplemental Figure 2, A–D), indicating that mTORC1 signaling is preferentially required for tumor endothelium.

### Low-dose RAD001 treatment increases the numbers and effector functions of TILs.

**Figure 4:**
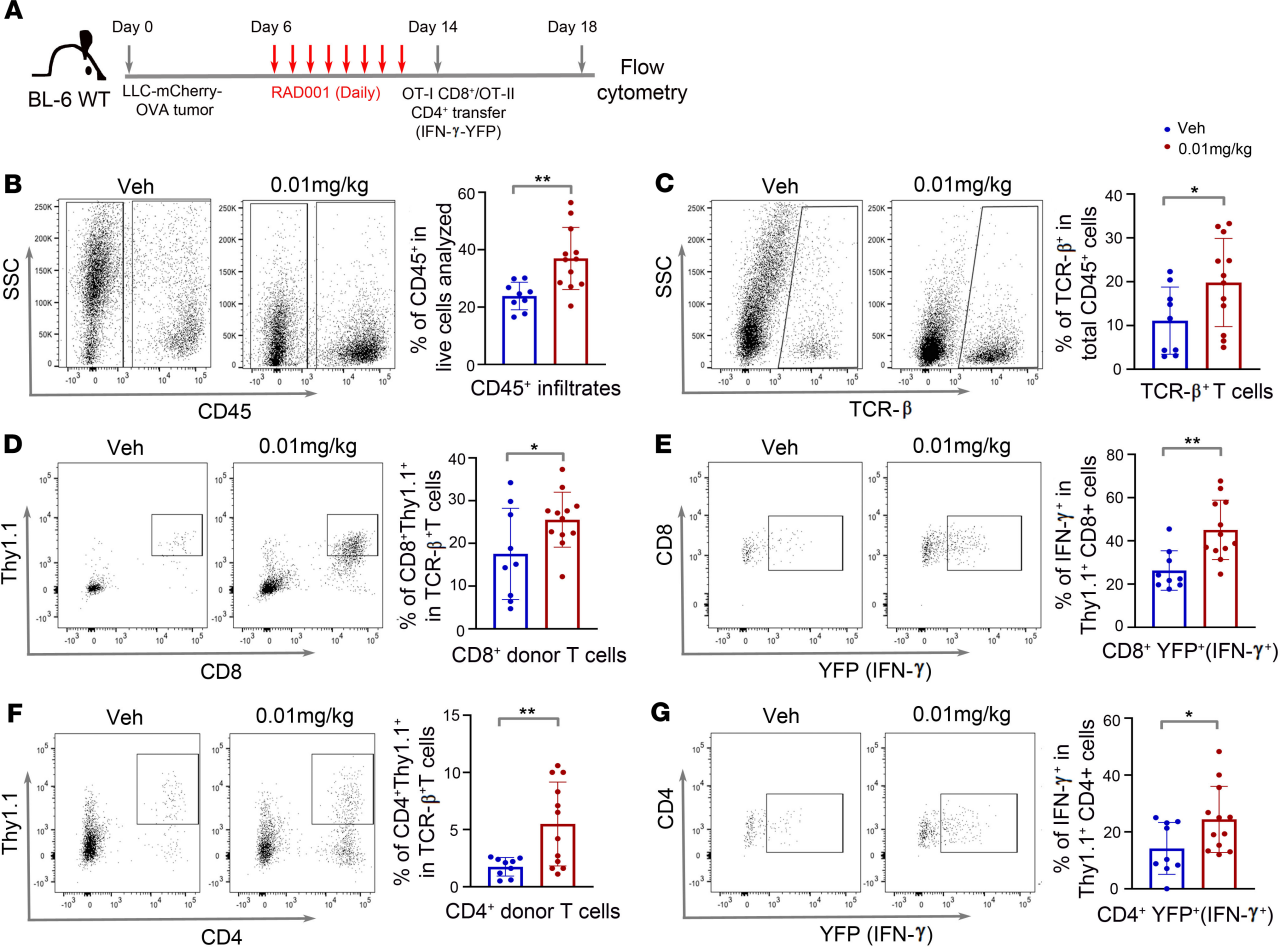
Low-dose RAD001 increases the numbers and effector function of infiltrating T lymphocytes. (**A**) Schematic diagram showing the experimental design with LLC-HRE-mCherry-OVA tumor cell implantation, RAD001 treatment, and adoptive T cell transfer. (**B** and **C**) Representative flow cytometric plots and quantification of tumor-infiltrating CD45^+^ immune cells (**B**) and TCRβ^+^ T cells (**C**) in LLC tumors treated with low-dose RAD001. (**D** and **E**) Representative flow cytometric plots and quantification of Thy1.1^+^CD8^+^ (**D**) and CD8^+^YFP^+^ (IFN-γ^+^) (**E**) donor T cells in LLC tumors treated with low-dose RAD001. (**F** and **G**) Representative flow cytometric plots and quantification of Thy1.1^+^CD4^+^ (**F**) and CD4^+^YFP^+^ (IFN-γ^+^) (**G**) donor T cells in LLC tumors treated with low-dose RAD001. All data are presented as mean ± SD. **P ≤ 0.01. *P ≤ 0.05, Student’s 2-tailed *t* test.

Functional vascular networks govern immune cell trafficking in untransformed tissues (17). Given that endothelial Raptor loss or mTORC1 inhibition improved vascular function within the tumor microenvironment, we next assessed leukocyte infiltration in LLC-HRE-mCherry-OVA tumors after low-dose RAD001 treatment (Figure 4A). Against the dogma of immune-suppressive function of RAD001, 0.01 mg/kg RAD001 low-dose treatment increased the numbers of infiltrating total CD45^+^ leukocytes and TCRβ^+^ T cells (Figure 4, B and C), consistent with its positive effect on vessel normalization (Figure 3, A–C). Importantly, there was a significant increase in numbers and effector function of adoptively transferred donor OT-I CD8^+^ T cells, as measured by percentages of OT-I CD8^+^ T cells (Figure 4D) and YFP^+^Thy1.1^+^CD8^+^ cells (Figure 4E), respectively (donor T cells expressing an IFN-γ–YFP reporter, refs. 28–30). Low-dose (0.01 mg/kg) RAD001 also enhanced infiltration of Thy1.1^+^CD4^+^ donor T cells (Figure 4F) and their activity, with an increase in YFP^+^ (IFN-γ^+^) Thy1.1^+^CD4^+^ T cells (Figure 4G). Together, these data suggest that low-dose RAD001 increases TILs and enhances effector function of adoptively transferred T cells.

### Loss of endothelial Raptor/mTORC1 increases TILs and enhances cytotoxic effector functions of CD8^+^ T cells.

**Figure 5:**
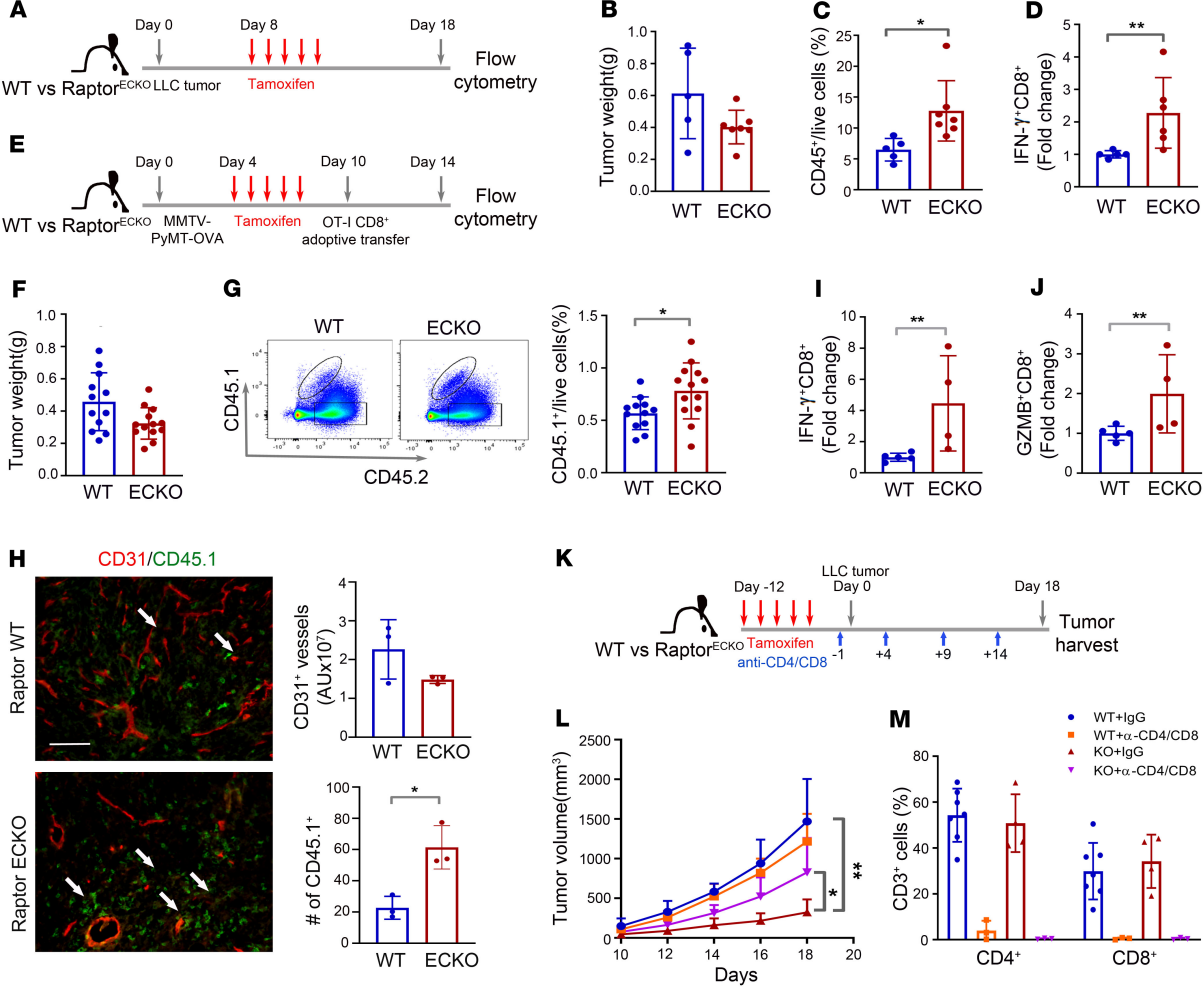
Raptor/mTORC1 loss in endothelium increases the numbers and effector function of tumor-infiltrating T cells. (**A**) Schematic diagram of experimental design with LLC tumor allograft. (**B**) Tumor weight at day 18 after implantation. Each dot represents a mouse. (**C** and **D**) Flow cytometric analysis of CD45^+^ (**C**) and IFN-γ^+^CD8^+^ (**D**) immune cells in WT and Raptor^ECKO^ tumors. (**E**) Schematic diagram of experimental design in MMTV-PyMT-OVA tumor orthotopic allograft. (**F**) Tumor weight at day 14 after implantation. (**G**) Flow cytometric analysis of donor (CD45.1) and recipient (CD45.2) CD45^+^ immune cells in WT and Raptor^ECKO^ tumors. Donor CD45.1^+^ cells were quantified and shown on the right. (**H**) Immunofluorescence images of CD45.1 (green) CD8 OT-I donor T cells costained with CD31 (red) in PyMT tumors. Numbers of CD45.1^+^ cells were quantified. Arrows indicate CD45.1^+^ donor T cells in the LLC tumor. Scale bar: 100 μm. (**I** and **J**) Flow cytometric analysis of IFN-γ (**I**) and GZMB (**J**) in CD8^+^ T cells in WT and Raptor^ECKO^ PyMT-OVA tumors. (**K**) Schematic diagram of experimental design in LLC tumors treated with anti-CD4 and anti-CD8 neutralizing antibodies. (**L**) Growth curves of LLC tumors on WT control and Raptor^ECKO^ mice after T cell depletion. *n* = 3–7 mice per group. Two-way ANOVA. (**M**) T cell depletion was confirmed by flow cytometric analysis. Unless indicated, all data are presented as mean ± SD. **P ≤ 0.01. *P ≤ 0.05, Student’s 2-tailed *t* test.

To verify the impact of endothelial mTORC1 on tumor immune response, we next performed a tumor size–matched experiment by implanting LLC tumors in tamoxifen-naive Raptor^WT^ or Raptor^ECKO^ mice and growing tumors for 8 days, at which point tamoxifen was administered to induce Cre^ER^ activity within vascular ECs (Supplemental Figure 3A). Tumors were harvested 10 days after initial tamoxifen treatment (Figure 5A), when tumor weight was not significantly different between WT and Raptor^ECKO^ mice (Figure 5B). Consistent with our finding in RAD001-treated mice (Figure 4B), significantly increased CD45^+^ leukocyte populations were observed from tumors grown in tamoxifen-treated Raptor^ECKO^ tumors (Figure 5C). More detailed immunophenotyping of tumor leukocyte populations revealed no consistent changes in the proportion of CD19^+^ B cells, F4/80^+^ macrophages, CD11b^+^Ly6G^+^ myeloid cells, NK cells, or CD4^+^CD25^+^FoxP3^+^ regulatory T cells associated with loss of Raptor from tumor ECs (Supplemental Figure 3C). Analysis of the tumor CD3^+^ T cell populations revealed that proinflammatory CD8^+^IFN-γ^+^ T cells were significantly increased in Raptor^ECKO^ tumors (Figure 5D), and CD4^+^IFN-γ^+^ T helper cells were moderately increased (Supplemental Figure 3E). These data suggest that Raptor/mTORC1 loss from tumor ECs increases TILs, particularly those expressing proinflammatory markers. Therefore, mTORC1-mediated activity contributes to tumor vessel dysfunction, which in turn may alter the recruitment and phenotype of TILs.

To test whether our findings apply to other tumor types, we used allografted *MMTV-PyMT* mouse mammary tumor cells engineered to express OVA, referred to as PyMT-OVA (Figure 5E). PyMT-OVA tumors were implanted into the tamoxifen-naive Raptor^ECKO^ or Raptor^WT^ mice. Four days following tumor implantation, mice were treated with tamoxifen to delete Raptor expression in the endothelium, followed by adoptive transfer of preactivated OT-I CD8^+^ T cells at day 10, and tumors were collected on day 14 (Supplemental Figure 3B and Figure 5F). Flow cytometric analysis and immunofluorescent detection of OT-I CD8^+^ T cells (gated or stained as CD45.1^+^) in PyMT-OVA tumors demonstrated a marked increase in tumor antigen–specific, exogenously delivered OT-I CD45.1^+^CD8^+^ T cells within the tumor parenchyma of PyMT-OVA tumors grown in Raptor^ECKO^ mice (Figure 5, G and H). Further, proinflammatory CD8^+^IFN-γ^+^ T cells were increased in PyMT-OVA tumors grown in Raptor^ECKO^ mice (Figure 5I), as were CD8^+^Granzyme B^+^ (GZMB) T cells (Figure 5J). Several other leukocyte populations occurred with similar frequency in Raptor^WT^ and Raptor^ECKO^ tumors, although CD4^+^ T cells were moderately increased in Raptor^ECKO^ samples (Supplemental Figure 3, D and F). Together, these data suggest that loss of mTORC1 signaling within the tumor vasculature may permit greater TIL infiltration by T cells harboring proinflammatory markers, including those T cells specifically directed toward tumor antigens.

Because loss of Raptor/mTORC1 increased proinflammatory CD8^+^IFN-γ^+^ T cells (Figure 5D) and CD4^+^IFN-γ^+^ T helper cells (Supplemental Figure 3E) in LLC tumors, we next determined whether T lymphocytes contribute to tumor suppression seen in Raptor^ECKO^ mice. LLC tumor–bearing mice were treated with multiple doses of anti-CD4 or anti-CD8 neutralizing antibodies, the combination of anti-CD4/anti-CD8 antibodies, or with isotype-matched control IgGs (Figure 5K). Consistent with our finding in Figure 2C, tumors in IgG-treated Raptor^ECKO^ mice grew at a substantially reduced rate as compared with tumors in IgG-treated Raptor^WT^ mice (Supplemental Figure 3G). Despite reduction of CD8^+^ T cells by anti-CD8 antibody treatment (Supplemental Figure 3H), tumor growth in either Raptor^WT^ or Raptor^ECKO^ mice was similar upon CD8 depletion as compared with what was seen in IgG-treated tumors (Supplemental Figure 3G). Similarly, anti-CD4 antibody alone did not significantly influence tumor growth in Raptor^ECKO^ mice, although CD4^+^ T cells were neutralized (Supplemental Figure 3, I and J). Interestingly, the combined depletion of CD8 and CD4 together partially restored tumor growth (Figure 5, L and M), suggesting that T lymphocytes, at least in part, play a major role in limiting tumor growth in Raptor^ECKO^ mice. These data support the hypothesis that vascular mTORC1 inhibition enhances tumor infiltration by T cells, which actively limit tumor growth.

### Endothelial Raptor/mTORC1 deficiency upregulates GM-CSF in Raptor^ECKO^ tumors.

**Figure 6:**
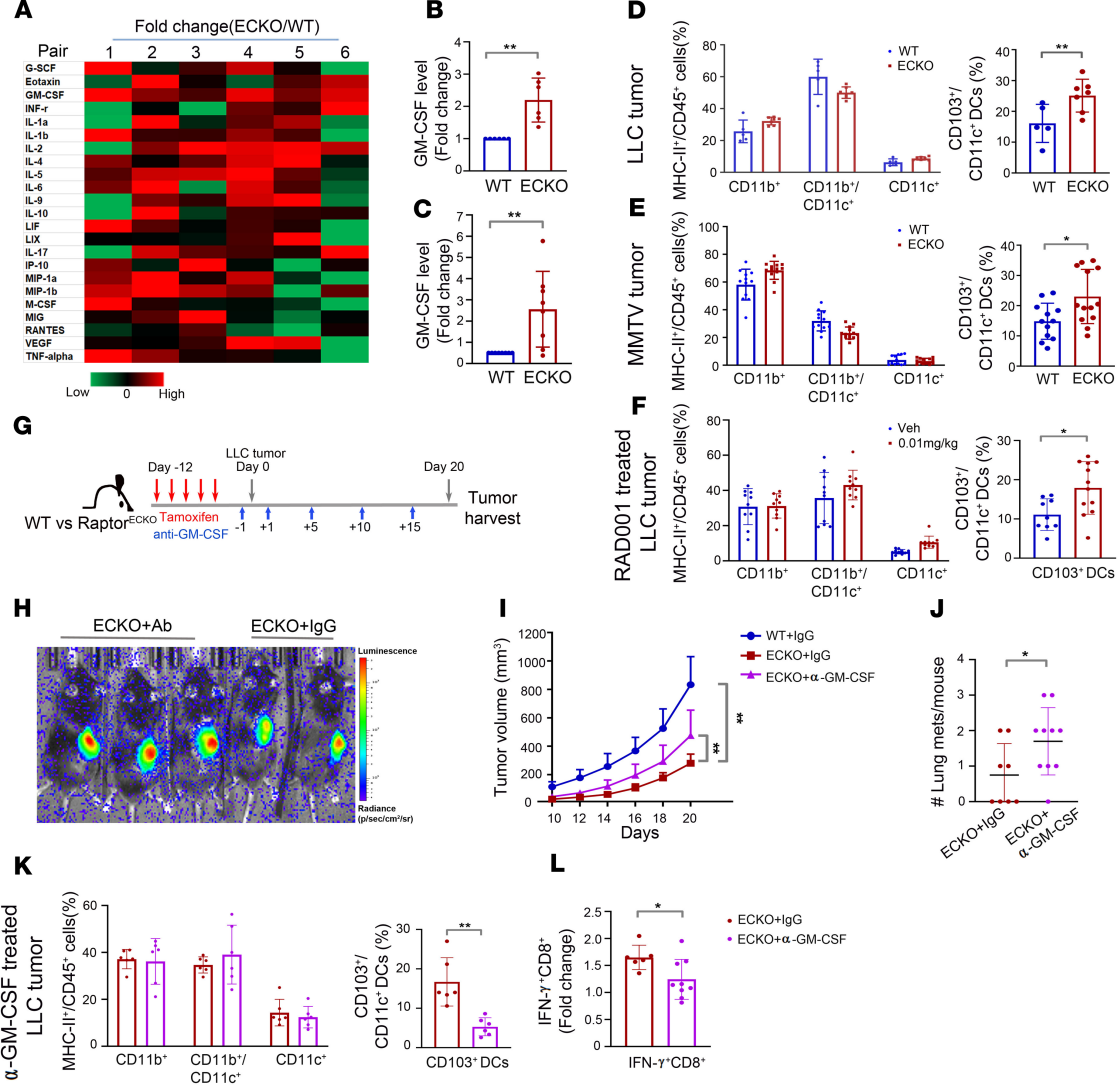
GM-CSF is required for increase in CD103^+^ DCs and IFN-γ^+^CD8^+^ T cells in Raptor^ECKO^ tumors. (**A**) Heatmap showing relative expression levels of indicated chemokines/cytokines in LLC tumors harvested from 6 sex-matched littermate pairs of WT and Raptor^ECKO^ mice. Red indicates higher expression while green indicates lower expression in Raptor^ECKO^ tumors over WT control. (**B**) Quantification of GM-CSF expression in Raptor^ECKO^ tumors and WT control tumors from Luminex analysis. (**C**) GM-CSF ELISA on independent LLC tumor lysates to verify elevated GM-CSF expression in Raptor^ECKO^ tumors. *n* = 8 mice per group. (**D**–**F**) Flow cytometric analysis of CD11b^+^, CD11b^+^CD11c^+^, or CD11c^+^ immune cells and CD103^+^ DCs in WT and Raptor^ECKO^ tumors from the LLC model (**D**) and PyMT model (**E**) or in LLC-HRE-mCherry-OVA tumors treated with low-dose RAD001 (**F**). (**G**) Schematic diagram showing the experimental procedure with GM-CSF neutralizing antibody treatment in the LLC model. (**H**) Representative image of bioluminescence signal from LLC tumors on Raptor^ECKO^ mice treated with anti–GM-CSF or IgG control on day 20 postimplantation. (**I**) Growth curves of LLC tumors on WT mice treated with IgG and Raptor^ECKO^ mice treated with anti–GM-CSF or IgG isotype antibodies. *n* = 8–11 mice per group. **P ≤ 0.01. Two-way ANOVA. (**J**) Quantification of metastatic foci on the surface of lungs harvested in **I**. (**K** and **L**) Flow cytometric analysis of CD11b^+^, CD11b^+^CD11c^+^, or CD11c^+^ immune cells and CD103^+^CD11c^+^ DCs (**K**) and IFN-γ^+^CD8^+^ T cells (**L**) in tumors treated with IgG control and tumors treated with anti–GM-CSF. Unless indicated, all data are presented as mean ± SD. **P ≤ 0.01. *P ≤ 0.05, Student’s 2-tailed *t* test.

To determine potential molecular mechanisms by which endothelial Raptor/mTORC1 may dysregulate TIL activity, LLC tumor lysates from Raptor^ECKO^ mice and their Raptor^WT^ sex-matched littermate controls (6 KO-WT pairs) were screened for cytokine and chemokine production by Luminex multiplex assay (Figure 6A). Of 32 tested cytokines/chemokines (Supplemental Table 1), GM-CSF was significantly elevated in Raptor^ECKO^ tumors across all 6 littermate pairs of WT and Raptor^ECKO^ tumors (Figure 6B). ELISA on independent tumor lysates revealed a nearly 5-fold increase in GM-CSF protein levels in Raptor^ECKO^ tumors compared with Raptor^WT^ tumors, confirming elevated GM-CSF protein levels in tumors grown in mice lacking vascular Raptor/mTORC1 (Figure 6C). GM-CSF, among other functions, is known to stimulate DCs, promoting antigen presentation by DCs to both B and T cells (35). Indeed, GM-CSF has been used as an adjuvant to enhance vaccine efficacy in clinical trials to promote antitumor immunity in melanoma and pancreatic cancer (36, 37). Among the many subtypes of DCs, tumor-resident CD103^+^ DCs support T cell priming and effector T cell trafficking (38–40). Consistent with these findings, the percentage of DCs that were CD103^+^ was significantly elevated in LLC tumors (Figure 6D) and MMTV-PyMT-OVA tumors (Figure 6E) grown in Raptor^ECKO^ mice, as well as in LLC-OVA tumors treated with 0.01 mg/kg of RAD001 (Figure 6F), though no consistent changes in the overall numbers of MHC-II^+^CD11c^+^ DCs were seen in tumors grown in WT and Raptor^ECKO^ mice or treated with vehicle and a low dose of RAD001.

To determine if increased CD103^+^ DCs are dependent on elevated GM-CSF in Raptor^ECKO^ mice, LLC tumors were implanted into tamoxifen-treated mice, and the tumor-bearing mice were treated with GM-CSF neutralizing antibodies or isotype-matched control IgG (Figure 6G). Depletion of GM-CSF partially restored defects of tumor growth (Figure 6, H and I) and lung metastasis in Raptor^ECKO^ mice (Figure 6J), compared with Raptor^ECKO^ animals treated with IgG control. Importantly, GM-CSF depletion significantly reduced tumor-infiltrating CD103^+^ DCs in Raptor^ECKO^ mice with no changes in the total CD11c^+^ DCs (Figure 6K). Anti–GM-CSF also decreased the percentage of IFN-γ^+^CD8^+^ T cells (Figure 6L) in Raptor^ECKO^ tumors. Taken together, these results suggest that GM-CSF is required for increased CD103^+^ DCs and enhanced cytotoxic CD8^+^ T cell activity in Raptor^ECKO^ tumors.

### Analysis of mTORC1 signaling, vessel normalization, GM-CSF, and immune markers in human cancer data sets.

**Figure 7:**
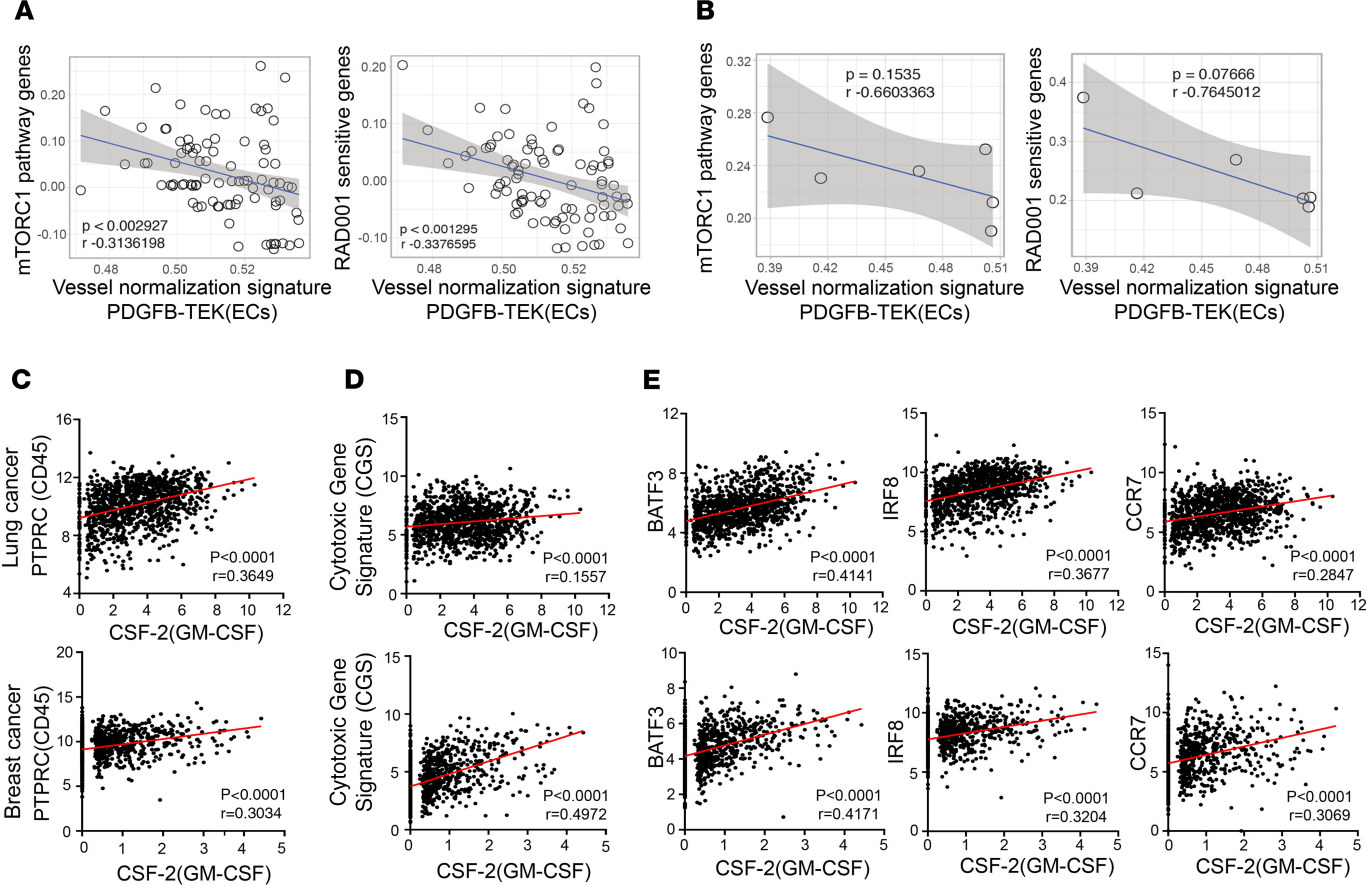
Correlations between vessel normalization markers and mTORC1-mediated signaling, as well as GM-CSF and immune markers in human tumor samples. (**A** and **B**) Correlation between vessel normalization markers and mTORC1-mediated signaling using non–small cell lung cancer (NSCLC, GSE127465) (**A**) or triple-negative breast cancer (TNBC, GSE118390) (**B**) scRNA-Seq data sets. The *x* and *y* axes represent ssGSEA enrichment scores of indicated gene sets in ECs. Each point represents a single EC. (**C** and **D**) Correlations between *CSF-2* (GM-CSF) transcript levels and levels of an immune cell marker *PTPRC* (CD45) (**C**) and a cytotoxic activity signature (an average expression of *IFNG*, *GZMB*, and *PRF1*) (**D**) in lung tumors (top) and breast tumors (bottom). (**E**) Correlations between *CSF-2* (GM-CSF) transcript levels and levels of *BATF3*, *IRF8*, and *CCR7* in lung tumors (top panels) and breast tumors (bottom panels). *BATF3*, *IRF8*, and *CCR7* are “signature genes” for the human CD103^+^ DC population. The *x* and *y* axes are log_2_(mRNA) expression using the Lung Cancer (LUNG) data set (*n* = 1129) and the Breast Cancer (BRCA) cohort (*n* = 1218) from TCGA. Each dot represents a single tumor sample. Pearson correlation coefficient (*r*) and 2-tailed *P* values are shown.

Because our data suggested that endothelial mTORC1 signaling is a key regulator of vascular dysfunction in tumors, contributing to tumor growth and immune evasion, we assessed possible correlations that may exist between a vessel normalization gene signature and mTORC1-mediated gene signatures using single-cell RNA-Seq data derived from patients with non–small cell lung cancer (NSCLC) or triple-negative breast cancer (TNBC) (41, 42). We focused on tumor-associated ECs that were already defined in these studies and performed single-sample gene set enrichment analysis (ssGSEA). Enrichment scores of endothelial *PDGFB* and *TEK/Tie2*, 2 genes known to regulate recruitment and proper integration of pericytes to blood vessels (15, 43, 44), were used as a vessel normalization signature. mTORC1 activity was assessed by using the mTORC1 pathway gene set (REACTOME) and the RAD001-sensitive gene set (CREIGHTON, which is defined as those that are upregulated upon overexpression of AKT and subsequently downregulated by RAD001, an mTORC1 inhibitor) listed in Supplemental Table 2. Analyses of correlations between the vessel normalization signature and the mTORC1-mediated signaling pathway gene set revealed that ECs with higher mTORC1 activity express fewer vessel normalization markers in both NSCLC (Figure 7A) and TNBC tumors (Figure 7B). Consistently, expression of RAD001-sensitive genes also negatively correlated with the expression of normalization markers *PDGFB* and *TEK* in these ECs. These findings suggest that lower endothelial mTORC1 activity correlates with vessel normalization in human cancer.

Given our finding that upregulation of GM-CSF in Raptor^ECKO^ tumors increased the proportion of CD103^+^ DCs and IFN-γ^+^CD8^+^ T cells, we next examined if GM-CSF has an impact on antitumor immunity in clinical lung (*n* = 1129) and breast cancer (*n* = 1218) data sets curated by The Cancer Genome Atlas (TCGA). Using *PTPRC* (CD45) gene expression as a proxy for total tumor leukocyte content, we identified a positive correlation between *CSF-2* (GM-CSF) and *CD45* in both lung and breast cancer patients (Figure 7C). To assess the level of cytotoxic immune cell activity in tumors, we averaged expression of 3 genes, IFN-γ (*IFNG*), *GZMB*, and perforin (*PRF1*), as a cytotoxic signature, similar to what was described previously (45, 46). Notably, higher *CSF-2* (GM-CSF) expression in both human lung and breast cancer samples positively correlated with the cytotoxic gene signature (Figure 7D). Further, CD103^+^ DCs are a distinct DC population that is uniquely dependent on IRF8 and Batf3 transcription factors and predominantly expresses CCR7 in human tumors, expression of which was used as a molecular proxy for CD103^+^ DCs in clinical tumor gene expression data sets (47, 48). Our analysis revealed a positive correlation between *CSF-2* (GM-CSF) and *IRF8*, *BATF3*, or *CCR7* in both lung and breast cancer patient cohorts (Figure 7E). Taken together, these findings suggest strong links between EC/mTORC1 signaling and vessel normalization and between GM-CSF and antitumor immunity in human cancers.

## Discussion

Tumor blood vessel normalization has been increasingly recognized to play critical roles in improving tissue perfusion, decreasing tumor hypoxia, and reducing vessel leakage, which lead to better drug delivery, improved antitumor immunity, and inhibition of tumor metastasis (11, 49). The mechanisms of vessel normalization, however, are not completely understood, such that actionable molecular targets for therapeutic tumor normalization remain elusive. Here we report the discovery that Raptor/mTORC1 knockout in vascular ECs decreases tumor hypoxia, growth, and metastasis through vessel normalization. Interestingly, EC-specific Raptor/mTORC1 loss also enhanced recruitment and activation of TILs, in part through GM-CSF–dependent activation of CD103^+^ DCs. Further, analyses of clinical tumor gene expression data sets demonstrated a negative correlation between vascular normalization markers and mTORC1 target gene signatures and a positive correlation between GM-CSF levels and molecular markers of T cell and DC activation. Notably, mTORC1 inhibition using low-dose RAD001 normalized tumor blood vessels, increased TILs, and improved adoptive T cell therapy. Although further studies are necessary to investigate whether tumor blood vessel normalization plays a causal role in the enhanced antitumor immunity in our model systems, these studies demonstrate that endothelial mTORC1 is an actionable target for tumor vessel normalization, which could offer supportive therapy for both traditional cancer treatments and emerging immunotherapies.

It is well accepted that T lymphocytes play a critical role in mediating antitumor immunity. The fact that depletion of T lymphocytes partially reversed the phenotype in Raptor^ECKO^ mice supported our model that inhibition of mTORC1 signaling in vascular ECs enhances antitumor immune response through a T cell–dependent mechanism (Figure 4 and Figure 5). The mechanisms by which tumor vessel normalization enhances TIL infiltration are likely to be mediated through chemokines and adhesion molecules, such as CXCL9/10 and ICAM1/E-selectin, respectively (13, 17, 50). Increased perfusion in normalized vessels could also take oxygen and nutrients (e.g., glucose and amino acids) into the tumor microenvironment for optimal T cell activation and effector function (51, 52). Further, tumor metabolites such as lactate and fatty acid have been shown to induce T cell apoptosis, inhibit effector T cell function, and induce M2 macrophage polarization (51, 52). More efficient removal of these metabolites by normalized vessels would release the inhibition of T cell function imposed by these tumor metabolites. Thus, our finding that mTORC1 inhibition within tumor vessel ECs supports tumor vessel normalization provides a foundation on which to begin studying these questions.

We show that Raptor/mTORC1 loss in endothelium leads to increased GM-CSF, which enhances antitumor immunity (Figure 6). However, GM-CSF has a dichotomous role in antitumor immunity. As a hematopoietic growth factor, GM-CSF controls the differentiation of the myeloid lineage and functions as an immunomodulator to stimulate DC maturation and granulocyte/macrophage activity. Studies on preclinical models show that GM-CSF increases exogenous antigen presentation capacity of CD103^+^ DCs (53–55) and promotes M1 macrophage polarization and T cell activation (56). Mice lacking GM-CSF or its receptor fail to establish protective immunity against a range of microbes and self-antigens (57–59). In this context, GM-CSF has been used as a cancer vaccine adjuvant to enhance the recruitment, maturation, and function of DCs in preclinical models and human clinical trials (35–37, 60, 61). In contrast to its immune-promoting roles, GM-CSF derived from tumors has been reported to drive the development of myeloid-derived suppressor cells (MDSCs) that suppress antitumor T cell functions (62, 63). In our system, Raptor^ECKO^ increased GM-CSF expression and CD11c^+^CD103^+^ DC infiltration in tumors (Figure 6, A–E) but did not consistently increase the infiltration of CD11b^+^Ly6G^+^ (Gr1^+^) cells, a population commonly defined as MDSCs, in the tumor microenvironment (Supplemental Figure 3, C and D), suggesting that the primary role of GM-CSF in Raptor^ECKO^ tumors is to promote CD103^+^ DC functions.

The tumor microenvironment (TME) is a diverse landscape, containing a variety of cell types, including tumor cells, immune cell populations (lymphocytes, macrophages, NK cells, DCs, MDSCs), fibroblasts, and ECs, among others (64, 65). The current study focuses on T cells and CD103^+^ DCs, but we cannot exclude the possibility that other immune populations, such as NK cells, may also play a role in the antitumor immune response in Raptor^ECKO^ tumors. Loss of endothelial Raptor/mTORC1 led to an increase in infiltrating NK and CD11b^+^Ly6G^+^ myeloid cell populations in LLC tumors (Supplemental Figure 3C). In addition to their intrinsic cytotoxic activity, NK cells have also been shown to produce CCL5 and XCL1 to recruit conventional DC1s, which express CD103^+^, to promote antitumor immunity (66). CD11b^+^Ly6G^+^ are markers used to define granulocytic MDSCs or neutrophils, and the latter population is able to inhibit tumor growth by suppressing IL-17 expression at the early stage of tumorigenesis (67). Furthermore, GM-CSF was reported to promote M1 macrophage polarization, which could also promote antitumor immunity (56, 68). However, increased infiltration of NK and CD11b^+^Ly6G^+^(Gr-1^+^) cells in Raptor^ECKO^ tumors was not seen in the MMTV-PyMT-OVA model (Supplemental Figure 3D), suggesting that endothelial Raptor/mTORC1 has a diverse contribution to the TME depending on tumor type. Nevertheless, the mechanism of the enhanced antitumor immune response in Raptor^ECKO^ tumors may involve multiple immune populations. Future work is needed to address the effect of Raptor/mTORC1 loss in endothelium on other immune cell populations.

Tumor immunotherapies have achieved dramatic success in clinical oncology. However, the majority of patients do not respond to such treatments. Because antitumor clinical responses rely on T cells that recognize and kill cancer cells, recruitment of such cancer-fighting T cells into the tumor parenchyma is paramount (69). Given the critical roles of blood vessels in regulating leukocyte trafficking in normal tissue, one likely mechanism of immune evasion is the abnormal tumor vasculature (4). Thus, normalization of tumor blood vessels holds the promise of increasing TILs in “immune-excluded” tumors. The discovery that loss of mTORC1 activity increases TILs opens up a new avenue of driving T cells into the tumor parenchyma. mTORC1 inhibitors, such as RAD001/everolimus, are FDA-approved drugs, but at a therapeutic dose, rapalogs inhibit the immune system. However, at low doses (~100-fold lower than the doses approved for organ transplant and oncology patients), partial inhibition of mTORC1 activity in clinical trials enhanced immune function and decreased infection rates in elderly patients (26, 27). Consistent with clinical trial results, we have found that low-dose RAD001 induces normalization of tumor blood vessels while increasing TILs (Figure 3 and Figure 4). Because RAD001 is a clinically approved drug, results from our studies can be quickly translated into clinical trials to improve both tumor sensitivity to immune checkpoint inhibitors and adoptive T cell therapy.

## Methods

Further information can be found in Supplemental Methods.

### Mouse models.

All mice used in this study were immunocompetent and housed in a nonbarrier animal facility. Raptor^fl/fl^ (C57BL/6), *MMTV-PyMT* transgenic mammary tumor model (C57BL/6), OT-I (C57BL/6), OT-II (C57BL/6), CD45.1 (C57BL/6), and C57BL/6 mice were purchased from The Jackson Laboratory. CDH5-CreER^T2^ mice (C57BL/6) were originally generated in Ralf Adam’s laboratory (Max Planck Institute, Münster, Germany) and provided by Hong Chen (Boston Children’s Hospital, Harvard Medical School, Boston, Massachusetts, USA). Animals were genotyped for MMTV-PyMT, OT-I, OT-II, Cre, or floxed *Raptor* alleles using the primers listed in Supplemental Table 3. CD45.1^+/+^ alleles were confirmed by FACS analysis. To induce *Raptor* KO specifically in the endothelium, tamoxifen (T5648, MilliporeSigma) was reconstituted in sunflower seed oil (S5007, MilliporeSigma) at 15 mg/mL, and a dose of 2 mg/mouse was administered to 7-week-old mice by intraperitoneal injection for 5 consecutive days. The treated mice had a 1-week break before tumor implantation.

### Tumor models and treatment regimens.

The Lewis lung cancer–luciferase (LLC-Luc) tumor chunk (32) and MMTV-PyMT-OVA cell line were gifts from Chi-Ping Day and Glenn Merlino (National Cancer Institute at Frederick, Frederick, Maryland, USA) and from Washington University in St. Louis, respectively. The LLC-Luc tumor fragment was expanded subcutaneously in Raptor^fl/fl^ mice for 4 passages before it was used for the experiment. To generate LLC-HRE-mCherry reporter cells, LLC parental cells, provided by Barbara Fingleton (Vanderbilt University), were infected with lentiviral HRE-mCherry and sorted for mCherry^+^ cells after culture under hypoxia conditions. The LLC-HRE-mCherry cells were further transduced with viruses carrying pLVX-Hygro-IRES-OVA to generate the LLC-HRE-mCherry-OVA cell line. For the LLC allograft model, an LLC tumor chunk was cut into small pieces (~1 mm in diameter), and the fragments were implanted subcutaneously into the dorsal flanks of recipient mice. For metastasis studies with size-matched primary tumors, the primary LLC tumors were surgically resected at 500 mm^3^, and the mice were kept for an additional 12 days to assess lung metastasis. For the orthotopic breast tumor model, MMTV-PyMT-OVA cells (~1 × 10^6^ in 100 μL) were injected into the fourth mammary glands of female mice. Tumor volumes were measured at given time points. For the spontaneous breast tumor model, tamoxifen-treated female mice were assessed for tumor formation weekly by palpation beginning at 16 weeks. Animals were sacrificed at 28 weeks before tumor burden reached humane endpoints. Tumor volume was calculated using the following formula: volume = length × width^2^ × 0.52. Lungs were harvested at the end of the studies and surface metastatic lesions were quantified.

For GM-CSF depletion experiments, anti–GM-CSF neutralizing antibody (250 μg, clone MP1-22E9, catalog BE0259, Bio X Cell) or anti–rat IgG2a isotype control (250 μg, clone 2A3, catalog BE0089, Bio X Cell) were injected intraperitoneally (i.p.) the day before (day –1) LLC tumor implantation, followed by injections on days +1 and +5 and every 5 days until tumors were harvested. For T cell depletion experiments, anti-CD4 (clone GK1.5, catalog BE003-1, Bio X Cell), anti-CD8α (clone 53-6.7, catalog BE0004-1, Bio X Cell), a combination of anti-CD4 and anti-CD8α, or their corresponding isotype controls rat IgG2b (clone LTF-2, catalog BE0090, Bio X Cell) and rat IgG2a were injected (200 μg/animal, i.p.) on the day before (day –1) LLC tumor implantation and on days +4,+9, and +14 until tumors were harvested on day 18.

For mTORC1 inhibitor studies, LLC-HRE-mCherry-OVA cells (0.5 × 10^6^ in 100 μL) were injected subcutaneously into C57BL/6 recipient mice. Six days after tumor implantation, mice were treated with RAD001/everolimus at 0.01 mg/kg or 0.05 mg/kg or vehicle daily by i.p. injection for 8 days. On day 14, OVA peptide–activated OT-I CD8^+^ and OT-II CD4^+^ T cells were adoptively transferred to tumor-bearing mice, and tumors were harvested on day 18. Three hours before harvesting, a hypoxic probe, EF5 (10 mM in 5% glucose, 200 μL/mouse, University of Pennsylvania, Philadelphia, Pennsylvania, USA, http://www.hypoxia-imaging.org), was injected intravenously into the recipient mice for analysis of hypoxia in the tumor.

### Tumor hypoxia and blood vessel perfusion assay.

To assess tumor hypoxia, 1 × 10^6^ HRE-mCherry LLC cells were implanted subcutaneously into the dorsal flanks of tamoxifen-treated mice. Tumor samples were harvested on day 16. CD31 staining on cryosections was performed as described in Supplemental Methods. Hypoxia region was determined by assessing mCherry^+^ area per field in each tumor sample using ImageJ software (NIH). For vessel perfusion analysis, LLC tumor–bearing mice were injected intravenously with 100 μL of biotinylated Tomato Lectin (B1175, Vector Laboratories) 5 minutes before tumors were harvested and processed for CD31 staining. Streptavidin-conjugated Alexa Fluor 488 (S32354; Invitrogen, Thermo Fisher Scientific) was used to detect Lectin^+^ vessels. The perfused area was defined as a percentage of Lectin^+^CD31^+^ of the total CD31^+^ area using ImageJ.

### Luminex assay and GM-CSF ELISA.

After 7 days from the last tamoxifen treatment, LLC tumors were subcutaneously implanted into the treated mice and harvested on day 20. Tumor lysates were prepared using celLytic MT reagent (C3228, MilliporeSigma) with supplemental proteinase inhibitor cocktail (P8430, MilliporeSigma). Detection of cytokines in tumor lysates was performed according to instructions using the Milliplex MAP mouse multiplex assay kit (MCYTMAG-70K-PX32, MilliporeSigma) and a FLEXMAP3D instrument (Luminex) as described before (70). Two quality control samples and 5 standards were run on the same plate to determine assay consistency and cytokine concentrations, respectively. Expression of tested cytokines/chemokines in LLC WT and Raptor^ECKO^ tumors was normalized to protein concentrations and summarized in Supplemental Table 1. For GM-CSF ELISA, LLC tumors were harvested at day 20, and tumor lysates were prepared using celLytic MT reagent as described above. The level of GM-CSF in tumor samples was determined by using Mouse GM-CSF Quantikine ELISA kit (MGM00, R&D Systems, Bio-Techne) according to the manufacturer’s instructions.

### Adoptive T cell transfer.

To activate CD8^+^ T cells in vivo, NP16-OVA protein (1 mg/mL, N5051, Biosearch Technologies) was injected with Alum adjuvant (77161, Thermo Fisher Scientific), at 1:1 ratio into 10-week-old OT-I transgenic female mice (CD45.1) for 1 week. CD8^+^ T cells were isolated from spleens and lymph nodes of immunized CD45.1 OT-I transgenic mice by negative selection using mouse CD8α^+^ T Cell Isolation Kit (130-104-075, Miltenyi Biotec). MMTV-PyMT-OVA tumor cells were implanted into female WT or Raptor^ECKO^ (CD45.2) recipients as described above and allowed to grow for 3 days. On day 4, mice were treated with tamoxifen for 5 consecutive days to deplete Raptor in ECs. On day 10, 3 × 10^6^ activated OT-I CD8^+^ T cells purified from CD45.1 donors were adoptively transferred into the WT or Raptor^ECKO^ tumor-bearing mice. Mice were sacrificed on day 14, and tumor-infiltrating immune cells were analyzed using flow cytometry, as described in Supplemental Methods. For RAD001/everolimus studies, splenocytes and lymph node cells were isolated from CD45.1 OT-I or CD45.1 OT-II mice and activated in vitro with OVA peptide SIINFEKL (1 μg/mL, vac-sin, InvivoGen) or peptide 323-339 (1 μg/mL, RP1060, GenScript) with the presence of IL-2 (20 U/mL, RO23-6019, Hoffmann-Roche) for 48 hours. Activated OT-I CD8^+^ T cells were isolated by negative selection as described above. Activated OT-II CD4^+^ T cells were negatively selected by using a mixture of anti-CD8 (130-117-044; Miltenyi Biotec) and anti-B220 (120-000-299; Miltenyi Biotec) microbeads. A combination of purified CD8^+^ and CD4^+^ T cells (1 × 10^6^ per population) was used for adoptive transfer.

### Analysis of single-cell RNA-Seq and TCGA data sets.

Single-cell RNA-Seq data sets derived from TNBC (GSE18390) and NSCLC (GSE127465) patient studies (41, 42) were used for correlation analyses in ECs. ssGSEA was performed on previously identified ECs to calculate gene set enrichment scores using GSVA (v1.28.0) in R using single-cell expression matrix (71). The gene set containing *PDGFB* and *TEK* was defined as “vessel normalization signature” in this study based on their critical roles in pericyte recruitment (44). The gene sets of “mTORC1-mediated signaling” and “Creighton_akt1 _signaling _via_mtor_dn” were chosen from REACTOME and chemical and genetic perturbations (72), respectively, under the C2 collection of Molecular Signatures Database and defined as “mTORC1 pathway genes” and “RAD001 sensitive genes” here. Genes used in these sets to calculate enrichment scores are listed in Supplemental Table 2. The Pearson product moment correlation was performed to determine a linear association between mTORC1 activity and vessel normalization in ECs. The *x* and *y* axes represent ssGSEA enrichment scores of indicated gene sets. Each data point represents a single EC.

TCGA BRCA database (data set ID: TCGA.BRCA.sampleMap/HiSeqV2, version 2017-10-13, *n* = 1218) and LUNG database (data set ID: TCGA.LUNG.sampleMap/HiSeqV2, version 2017-09-08, *n* = 1129) were used for correlation analysis between *CSF-2* (GM-CSF) and immune cell markers in the context of human tumors. Immune cytotoxic activity was calculated as an average expression of 3 genes, *IFNG*, *GZMB*, and *PRF1*, similar to what was performed in several previous studies (45, 46). The abundance of CD103^+^ DCs was determined by expression levels of *IRF8*, *Batf3*, and *Ccr7*, 3 “signature genes” that were reported previously to indicate CD103^+^ DC population in human tumors (47, 48). Scatterplots were generated using Prism to identify correlation profiles between log_2_(mRNA) expression of GM-CSF and immune cell makers in tumor samples. Each data point represents a single patient. Linear regression and Pearson correlation were determined using GraphPad Prism software.

### Statistics.

All plots and statistical analysis were completed using GraphPad Prism software. For comparisons between 2 groups, unpaired 2-tailed Student’s *t* tests were performed as indicated. For multiple comparisons, 1- or 2-way ANOVA was performed with Tukey’s or Holm-Šidák multiple comparisons test. For comparison of survival curves, log-rank (Mantel-Cox) test and Gehan-Breslow-Wilcoxon test were performed. The heatmap was generated using Microsoft Excel software and displayed on a logarithmic scale with values normalized per row. Unless indicated in the figures, all data are presented as mean ± SD, and *P* < 0.05 was considered statistically significant.

### Study approval.

Animal care and experimental procedures were performed under protocols approved by Vanderbilt University’s IACUC.

## Author contributions

SW and JC conceptualized the project. SW, AR, ES, SC, DMBS, DE QW, and SZ performed the experiments and analyzed the data. DD provided PyMT-OVA cells and the OVA expression construct. JN provided the HRE-mCherry construct. MMA and KTW helped on the Luminex assay. AR and MB provided helpful suggestions and scientific discussions. SW, RC, and JC wrote the manuscript.

## Supplementary Material

Supplemental data

## Version 1 08/06/2020

Electronic publication
