# Supplementary material for: Selective inhibition of mTORC1 in tumor vessels increases antitumor immunity
Source: JCI Insight. 2020 Aug 6;5(15):e139237. doi: 10.1172/jci.insight.139237 (PMC7455083; doi:10.1172/jci.insight.139237)
Supplement: Supplemental data [file jciinsight-5-139237-s141.pdf]

Supplemental Materials and Methods

Antibodies, immunofluorescence and immunohistochemistry. To prepare cryosections, tumor or normal tissue samples were immediately frozen in OCT compound (#50-363-579, Fisher Scientific) and kept at -80 degrees. 6-µm sections were cut on Leica Cryostat CM1950. Cryosections were fixed in 4% PFA for 30 minutes at room temperature followed by two washes with PBS and one wash with 0.3% Triton X-100 in PBS (5min/each wash). Samples were blocked with 2.5% goat serum (#G9023, Sigma-Aldrich) in PBS for 1 hour at room temperature. All primary antibodies were incubated overnight at 4 degrees, followed by secondary antibodies for 1 hour at room temperature. Tumor sections were mounted using SlowFade Diamond antifade reagent containing DAPI (#S36963, Molecular Probes). The following antibodies were used for immunofluorescence on cryosections: CD31 (1:150, #102501, Biolegend), α-SMA (1:150, #M085129-2, Dako), and FITC-CD45.1 (1:200, #35-0452, Tonbo Biosciences). Secondary antibodies used were goat-anti-rat Alexa Fluor 594 (# A11007; Invitrogen), goat-anti-rat Alexa Fluor 488 (#A11006, Invitrogen), goat-anti-mouse Alexa Fluor 488 (#A11001; Invitrogen) and goat-anti-mouse Alexa DyLight 680 (1:200, #35518; Invitrogen). Unless indicated, all secondary antibodies were used at 1:500 dilution. The α-SMA staining was performed using mouse on mouse Elite Peroxidase kit (#PK-2200, M.O.M, Vector Laboratories) to reduce background. Immunohistochemistry (IHC) was performed as described previously (1, 2), using biotin-conjugated anti-PCNA (proliferation cell nuclear antigen) (# 555567; Pharmingen). PCNA+ staining was quantified as percentage of PCNA$^+$ nuclei relative to total nuclei per section. Apoptosis assays were performed on paraffin sections using the Apoptag Red In Situ Apoptosis Detection Kit following the manufacturer’s protocol (#S7165, EMD Millipore). TUNEL$^+$ staining was quantified as the percentage of TUNEL$^+$ nuclei relative to total nuclei per section. Images were taken by an Olympus inverted fluorescence microscope and processed by using the Cellsens Dimension software program. Images of α-SMA staining on LLC-HRE-mCherry-OVA tumors were taken by a Nikon Eclipse Ti microscope and processed using NIS Elements AR software. 6-10 random fields (10x or 20x magnification) were taken per tumor section and analyzed using the NIH Image J.

Flow cytometry. Tumors were dissociated in RPMI-1640 containing 5% FBS, collagenase IA (1 mg/mL; #C9891, Sigma-Aldrich), hyaluronidase (0.1mg/ml; #H3506, Sigma-Aldrich) and DNase I (0.25 mg/mL; #DN25, Sigma-Aldrich) for 45 min at 37°C. To obtain single-cell suspensions, digested tissue was filtered through a 70-µm cell strainer and red blood cells were lysed using ACK lysis buffer (#RGF-3015, KD Medical). Flow cytometry data was obtained on a

BD Fortessa using BD FACS Diva software and analyzed using FlowJo software. Dead cells were excluded from analysis by staining with Ghost Dye Violet 510 (#13-0870, Tonbo). Following blocking with αCD16/32 (mouse Fc block; #70-0161, Tonbo Biosciences), extracellular staining was performed against the following markers: CD45, CD45.1, CD45.2, CD3e, CD4, CD8a, CD25, CD19, IA/IE, Ly6G (Gr-1), CD11b, CD11c, CD103, F4/80, and NK1.1 (Supplemental Table 4). For detection of intracellular cytokines interferon-γ (IFN-γ) and granzyme B (GZMB), $2x10^6$ cells were stimulated with phorbol 12-myristate 13-acetate (PMA) (50 ng/mL; #P8139, Sigma-Aldrich) and ionomycin (1 µg/mL; #I0634, Sigma-Aldrich) for 1 hour at 37°C, followed by GolgiPlug protein transport inhibitor (1:1000; #555029, BD) for additional 3 hours. Activated cells were permeabilized using Cytofix/Cytoperm solution kit (#554714, BD Biosciences) and stained with intracellular antibodies indicated in Supplemental Table 4. To detect FoxP3, extracellular stained cells were processed using the FoxP3/Transcription Factor Staining Kit (#TNB-0607, Tonbo Biosciences). To detect p-S6, $4.0x10^6$ cells were fixed in 100 µl of 4% PFA at 37°C for 15 min and permeabilized with ice-cold methanol for 30 min on ice. Cells were incubated with anti-p-S6 (Ser235-236) rabbit antibody (#2211S, Cell Signaling Technology) for 1 hour at 37 °C, followed by Fc block and subsequently stained with an antibody cocktail containing surface markers (CD45, CD31) and secondary antibody goat-anti-rabbit Alexa 647 (1:200; #A21244, Invitrogen). Isolated splenocytes or tumor cell suspensions were used for unstained, compensation, and fluorescence minus one (FMO) controls whenever appropriate. The markers used to define immune population are listed in Supplemental Table 5.

References

1. Amato, KR, Wang, S, Hastings, AK, Youngblood, VM, Santapuram, PR, Chen, H, et al. Genetic and pharmacologic inhibition of EPHA2 promotes apoptosis in NSCLC. *J Clin Invest.* 2014; 124:2037-2049.
2. Amato, KR, Wang, S, Tan, L, Hastings, AK, Song, W, Lovly, CM, et al. EPHA2 Blockade Overcomes Acquired Resistance to EGFR Kinase Inhibitors in Lung Cancer. *Cancer Res.* 2016; 76:305-318.

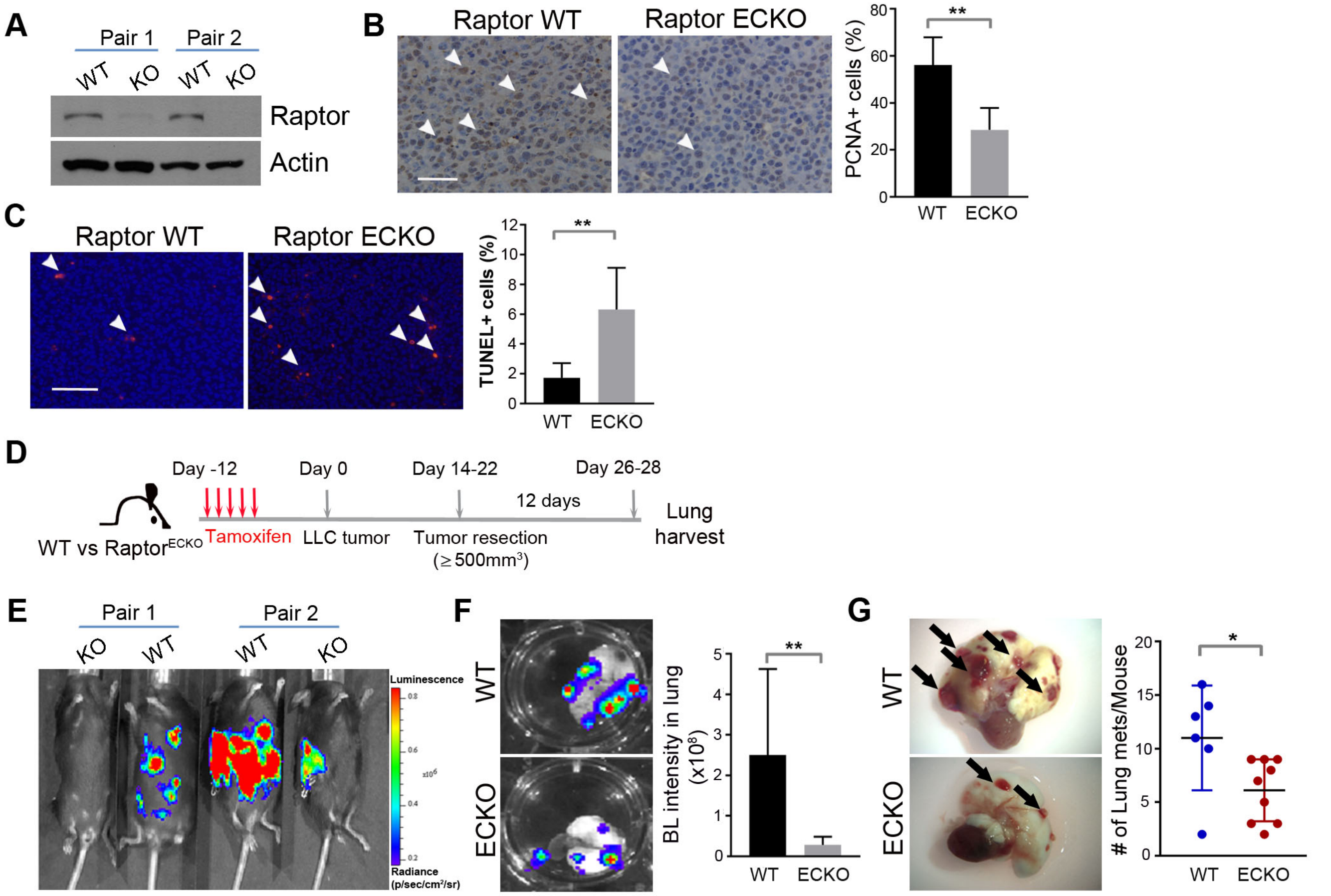

Supplemtnal Figure 1, related to Figure 2: Loss of Raptor/mTORC1 in endothelium decreases cell proliferation and increases apoptosis. **(A)** Western blotting to confirm deletion of Raptor alleles in primary pulmonary microvascular ECs isolated from Tamoxifen-treated WT and Raptor^{ECKO} mice. **(B)** Cell proliferation of LLC tumors was assessed by PCNA immunohistochemistry and was presented as the percentage of PCNA^{+} nuclei/total nuclei. Arrowheads indicate PCNA^{+} proliferating cells in tumors. n = 3-5 tumors per group. Scale bar: 50µm. **(C)** Apoptosis of LLC tumors was assessed by TUNEL staining. The apoptosis index is presented as the percentage of TUNEL+ nuclei/total nuclei. Arrowheads indicate apoptotic cells(red) in tumor. n = 3-5 tumors per group. Scale bar: 50µm. **(D)** Schematic diagram showing the experimental procedure of primary tumor resection to examine lung metastasis. LLC tumors on tamoxifen-treated mice were surgically removed once their volume reached 500mm^3. After surgery, mice were kept for additional 12 days to monitor lung metastasis. **(E)** Representative image of bioluminescence signal from metastatic LLC tumors on WT control and Raptor^{ECKO} mice. **(F)** Representative images and quantification of bioluminescence signal from LLC metastatic foci on the lungs harvested from WT control and Raptor^{ECKO} mice. n= 6-9 mice per group. BL: bioluminescence intensity. **(G)** Representative images and quantification of LLC metastatic foci on the surface of lungs harvested from WT control and Raptor^{ECKO} mice. Arrows indicate metastatic tumors on the lung surface. All data are presented as mean ± SD. **, $p \leq 0.01$. *, $p \leq 0.05$. Unpaired Student's t-test.

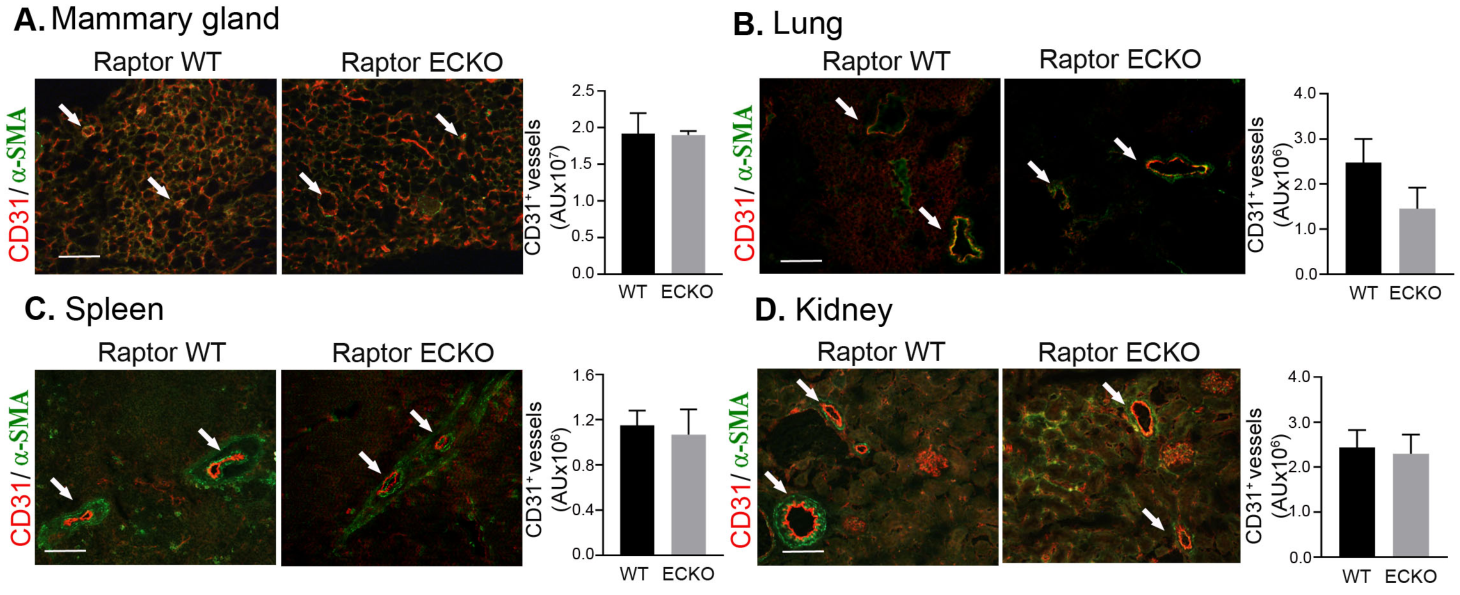

Supplemental Figure 2, related to Figure 3: Loss of Raptor/mTORC1 in tumor-free endothelium does not change vasculature in different tissues. WT and Raptor^{ECKO} mice were treated with tamoxifen for 5 consecutive days to induce EC-specific loss of *Raptor*. Once week after the last tamoxifen treatment, different tissues were harvested and vasculature was assessed by co-staining of CD31^{+} (red) and α-SMA (green) on **(A)** mammary glands, **(B)** lungs, **(C)** spleens, and **(D)** kidneys. Arrows indicate co-localization of CD31 and SMA in tissues. n = 3-5 mice per group. Scale bar: 100μm.

Wang *et al.*,Figure S3

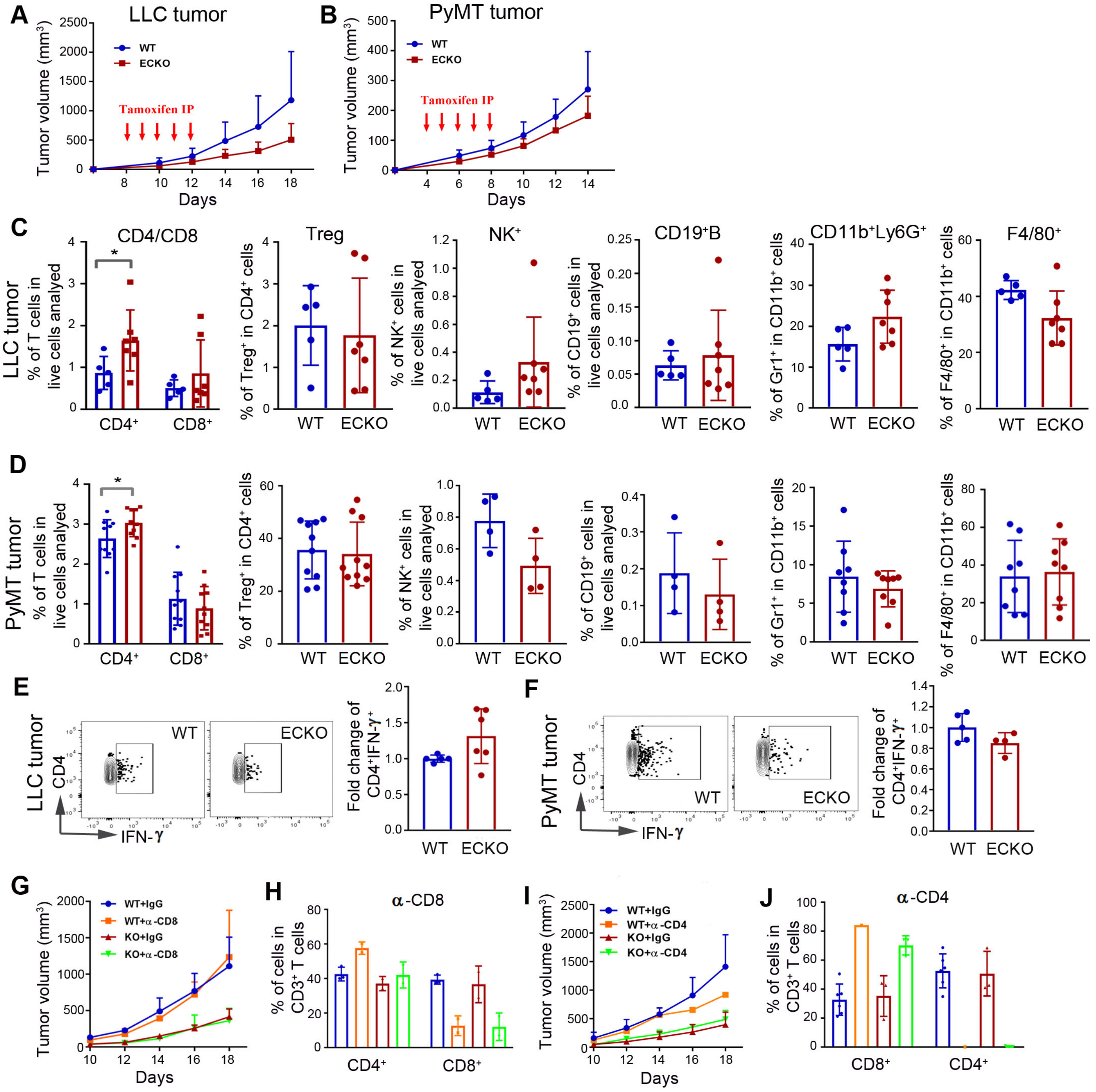

Supplemental Figure 3, related to Figure 5. Immune profiles of LLC and PyMT-OVA tumors from WT control and Raptor^{ECKO} mice. (A) Growth curves of LLC tumors on WT control and Raptor^{ECKO} mice. Tamoxifen treatment started on day 8 after tumor implantation. n=5-7 mice per group. **(B)** Growth curves of PyMT-OVA tumors on WT control and Raptor^{ECKO} mice. Tamoxifen treatment started on day 4 after tumor implantation. n=13-14 mice per group.**(C-D)** Flow cytometric analysis of T cells (CD4$^+$ and CD8$^+$), Treg cells (CD4$^+$CD25$^+$FoxP$^+$), NK cells (CD45$^+$NK1.1$^+$), B cells (CD45$^+$CD19$^+$), Ly6G+myeloid cells [CD11b$^+$Ly6G(Gr1)$^+$] and macrophages (CD11b$^+$F4/80$^+$) in LLC tumors **(C)** or MMTV-PyMT-OVA tumors **(D)** harvested from WT and Raptor^{ECKO} mice. **(E-F)** Representative flow cytometric plots and quantification of IFN-γ^+ CD4$^+$ T cells (Th1) in LLC tumors **(E)** and MMTV-PyMT-OVA tumors **(F)** harvested from WT and Raptor^{ECKO} mice. **(G-H)** Growth curves of LLC tumors on WT control and Raptor^{ECKO} mice with anti-CD8 treatment. CD8$^+$ T cell depletion was confirmed by flow cytometry in **(H)**. **(I-J)** Growth curves of LLC tumors on WT control and Raptor^{ECKO} mice with anti-CD4 treatment. CD4$^+$ T cell depletion was confirmed by flow cytometry in **(J)**. All data are presented as mean ± SD, **, $p \leq 0.01$. *, $p \leq 0.05$. Unpaired Student's t-test.

Supplemental Table 1: Cytokines in LLC tumors tested by Luminex. Cytokines (pg/µl) were standardized to protein concentration (µg/µl) in LLC tumors and expressed as pg/µg. Fold change was presented as mean ± SD. UN: undetectable, ST: saturated level. **, p<0.01. *, p< 0.05. Student's t-test.

Cytokines (pg/µg)	Pair 1		Pair 2		Pair 3		Pair 4		Pair5		Pair 6		Fold change (KO/WT)
	WT	KO	WT	KO	WT	KO	WT	KO	WT	KO	WT	KO	
**GM-CSF**	3.15	9.52	9.72	26.88	12.69	20.88	8.22	14.79	6.94	9.45	6.21	16.12	**2.20± 0.68 ** **
**IL-4**	0.16	0.35	0.07	0.25	0.05	0.24	0.06	0.07	0.04	0.08	0.11	0.09	**2.43± 1.58 * **
**IL-5**	0.62	0.68	0.57	0.75	0.45	0.50	0.69	0.86	0.45	0.57	0.79	0.71	**1.15± 0.15 * **
**G-CSF**	50.91	257.27	163.48	569.89	126.76	144.53	268.89	189.20	103.53	155.04	1241.81	172.12	2.00±1.88
**Eotaxin**	88.90	69.95	79.05	62.80	121.39	193.69	29.22	137.31	103.00	113.54	58.65	183.55	2.02±1.58
**INF-γ**	1.28	0.52	0.46	0.90	0.36	0.50	0.70	0.68	0.75	0.29	0.34	1.80	1.73±1.82
**IL-1α**	6.62	3.50	4.38	5.42	2.95	4.31	2.76	4.92	3.36	2.94	8.26	5.19	1.08±0.49
**IL-1β**	0.80	5.70	4.45	13.72	3.31	5.83	3.92	6.04	1.74	2.65	17.64	4.76	3.01±2.40
**IL-2**	0.50	0.13	0.18	0.26	0.16	0.24	0.21	0.26	0.12	0.18	0.15	0.21	1.19±0.47
**IL-3**	UN	UN	UN	UN	UN	UN	UN	UN	UN	UN	UN	UN	
**IL-6**	8.63	15.28	17.18	49.66	25.77	36.67	14.28	49.18	38.04	12.8	101.71	47.21	1.97±1.25
**IL-7**	0.26	0.19	0.29	0.19	UN	0.11	UN	0.60	0.11	0.06	UN	UN	
**IL-9**	39.35	19.45	22.04	39.25	22.30	45.00	34.43	48.62	15.75	18.03	54.60	33.41	1.24±0.61
**IL-10**	0.37	0.21	0.25	0.35	0.27	0.35	0.11	0.37	0.22	0.19	0.23	0.27	1.43±1.00
**IL-12**	UN	UN	UN	UN	UN	UN	UN	UN	UN	UN	UN	UN	
**IL-12p70**	UN	UN	UN	UN	UN	UN	UN	UN	UN	UN	UN	UN	

Cytokines (pg/μg)	Pair 1		Pair 2		Pair 3		Pair 4		Pair5		Pair 6		Fold change (KO/WT)
	WT	KO	WT	K	WT	KO	WT	KO	WT	KO	WT	KO	
LIF	5.35	20.08	17.60	25.13	22.82	27.12	20.47	32.27	17.51	16.91	56.35	19.30	1.54±1.17
IL-13	UN	UN	UN	UN	UN	UN	UN	UN	UN	UN	UN	UN	
LIX	UN	11.21	16.47	34.80	2.53	35.04	27.53	26.42	5.55	4.57	112.30	10.89	3.57±5.80
IL-15	0.80	1.35	UN	UN	UN	UN	UN	UN	UN	UN	UN	UN	
IL-17	0.62	0.19	0.21	0.24	0.22	0.24	0.14	0.21	0.14	0.17	0.15	0.26	1.18±0.50
IP-10	21.99	32.92	42.01	40.27	65.21	26.92	32.15	28.06	19.54	49.08	51.41	55.44	1.23±0.73
KC	624.23	ST	1151.90	ST	973.01	ST	1469.78	1335.07	860.53	1099.90	ST	1590.80	
MCP-1	1450.41	ST	ST	ST	1123.65	1128.99	ST	ST	ST	1089.96	2169.82	1256.04	
MIP-1α	22.65	27.87	28.19	37.78	41.00	37.79	27.47	40.14	28.99	29.97	52.17	37.06	1.11±0.30
MIP-1β	15.61	21.12	24.51	29.58	37.33	22.14	14.64	21.60	19.24	25.46	26.42	30.61	1.18±0.31
M-CSF	5.64	16.19	18.15	15.91	16.00	7.98	19.56	22.79	14.70	11.52	118.24	10.77	1.05±0.96
MIP-2	842.46	ST	ST	ST	1107.09	ST	ST	ST	ST	ST	ST	ST	
MIG	29.41	31.85	23.35	22.41	38.22	24.55	21.49	31.35	9.50	29.14	48.25	47.56	1.37±0.87
RANTES	2.75	1.99	2.11	1.20	3.59	1.18	1.04	1.00	1.20	1.61	2.30	2.47	0.84±0.37
VEGF	9.02	10.31	7.51	58.00	4.74	30.45	34.82	35.02	7.47	11.62	84.95	29.22	3.57±3.18
TNF-α	3.01	9.77	7.76	9.07	8.11	7.59	5.38	10.40	5.21	5.82	20.26	7.26	1.50±1.01

Supplemental Table 2: Gene sets used to perform correlation analysis in endothelial cells

**Gene sets in MsigDB**	**REACTOME_mTORC1_mediated_Signalling**	**CREIGHTON_AKT1_signalling_via_mTOR_DN**
	**mTORC1 mediated pathway**	**RAD001-sensitive genes**
**Collection**	**C2, CP**	**C2, CGP**
**Gene List**	AKT1S1	ALDOA
	EEF2K	ATP6V0C
	EIF4B	GPI
	EIF4E	KRT8
	EIF4EBP1	MIF
	EIF4G1	PAFAH1B3
	LAMTOR1	PPP2R1A
	LAMTOR2	RGL2
	LAMTOR3	YWHAB
	LAMTOR4	MRPS7
	LAMTOR5	ATP6V0B
	MLST8	PFKL
	MTOR	PPP4C
	RHEB	TOM1
	RPS6	TNFRSF12A
	RPS6KB1	ATP6AP1
	RPTOR	TSPAN1
	RRAGA	ATP6V1F
	RRAGB	DHCR7
	RRAGD	CIB1
	SLC38A9	GOT1
	YWHAB	

MSiDB: Molecular Signatures Database

Supplemental Table 3: Mouse genotyping primers

Primer name	Sequence	Concentration for [illegible]
Raptor-F	5’-CTCAGTAGTGGTATGTGCTCAG-3’	10μM
Raptor-R	5’-GGGTACAGTATGTCAGCACAG-3’	10μM
iCdh5-Cre-F	5’-TCCTGATGGTGCCTATCCTC -3’	10μM
iCdh5-Cre-R	5’-CGAACCTGGTCGAAATCAGT-3’	10μM
MMTV-PyMT-F	5’-GGAAGCAAGTACTTCACAAGGG-3’	25μM
MMTV-PyMT-R	5’-GGAAAGTCACTAGGAGCAGGG-3’	25μM
OT-I TCRα-F	5’-CAGCAGCAGGTGAGACAAAGT-3’	5μM
OT-I TCRα-R	5’-GGCTTTATAATTAGCTTGGTCC-3’	5μM
OT-I TCRβ-F	5’-AAGGTGGAGAGAGACAAAGGATTC-3’	20μM
OT-I TCRβ-R	5’-AAGGTGGAGAGAGACAAAGGATTC-3’	20μM
OT-II TCRα-F	5’-AAAGGGAGAAAAGCTCTCC-3’	10μM
OT-II TCRα-R	5-ACACAGCAGGTTCTGGTTC-3’	10μM
OT-II TCRβ-F	5’-GCTGCTGCACAGACCTACT-3’	10μM
OT-II TCRβ-R	5’-CAGCTCACCTAACACGAGGA-3’	10μM

Supplemental Table 4: Mouse Flow cytometry antibodies

Antibody	Clone	Dilution	Cat#	Company
APC-Cy7-CD45	30-F11	1:600	561037	BD Biosciences
APC-Cy7-CD45.1	A20	1:600	25-0453	Tonbo Biosciences
FITC-CD45.2	104	1:600	35-0454	Tonbo Biosciences
V450-CD45.2	104	1:600	75-0454	Tonbo Biosciences
PercP-Cy5.5-CD3e	145-2C11	1:300	561108	BD Biosciences
PE-Cy7-CD3e	145-2C11	1:600	552774	BD Biosciences
RedFluro710-CD8α	53-6.7	1:600	80-0081	Tonbo Biosciences
PE-CD8α	53-6.7	1:400	553033	BD Biosciences
APC-CD4	RM4-5	1:300	553051	BD Biosciences
PE-Cy5-CD4	GK1.5	1:600	55-0041	Tonbo Biosciences
V450-CD4	RM4-5	1:300	75-0042	Tonbo Biosciences
eFluor660-FoxP3	FJK-16s	1:100	50-5773-82	eBioscience
PE-CD25	PC61.5	1:400	12-0251-81	eBioscience
PE-Cy7-CD25	PC61.5	1:500	60-0251	Tonbo Biosciences
V450-IFN gamma	XMG1.2	1:100	75-7311	Tonbo Biosciences
PE-Granzyme B	NGZB	1:100	12-8898-80	Invitrogen
APC-F4/80	BM8	1:300	17-4801-82	eBioscience
RedFluro710-MHC Class II (IA/IE)	M5/114.15.2	1:1000	80-5321	Tonbo Biosciences
FITC-CD11b	M1/70	1:300	557396	BD Biosciences
PerCP-Cy5.5-CD11c	N418	1:300	65-0114	Tonbo Biosciences
PE-Cy7-CD11c	HL3	1:1000	561022	BD Biosciences
APC-CD103	2E7	1:300	17-1031-82	eBioscience
PerCP-Cy5.5-Ly6G(Gr1)	RB6-8C5	1:500	552093	BD Biosciences
PE-Cy7-CD19	1D3	1:1000	60-0193	Tonbo Biosciences
PE-NK1.1	PK136	1:300	557391	BD Biosciences
BV421-CD31	390	1:200	102423	Biolegend

Antibody	Clone	Dilution	Cat#	Company
APC-Thy1.1	HIS51	1:500	17-0900-82	eBioscience
PerCP-Cy5.5-TCR-β	H57-597	1:500	65-5961	Tonbo Biosciences

Supplemental Table 5: Markers used to define immune cell population

Immune cells	Markers
Lymphocytes	Ghost-CD45+
T cells	Ghost-CD45+CD3+
TCR-β T cells	Ghost-CD45+TCR-β+
OT-I CD8 donor cells	Ghost-CD45+TCR-β+Thy1.1+CD8+
OT-II CD4 donor cells	Ghost-CD45+TCR-β+Thy1.1+CD4+
CD4 T cells	Ghost-CD45+CD3+CD4+
CD8 T cells	Ghost-CD45+CD3+CD8+
Treg cells	Ghost-CD45+CD3+CD4+CD25+FoxP3+
NK cells	Ghost-CD45+CD3-NK1.1+
B cells	Ghost-CD45+CD19+
$Ly6G^{+}$ myeloid cells	Ghost-CD45+CD11b+Ly6G (Gr1) +
Macrophage	Ghost-CD45+ CD11b+Gr-F4/80+
$CD11c^{+}$ DC cells	Ghost-CD45+CD3-CD19-MHCII+CD11b-CD11c+
$CD103^{+}$ DC cells	Ghost-CD45+CD3-CD19-MHCII+CD11b-CD11c+CD103+
